# Light-regulated gene expression in Bacteria: Fundamentals, advances, and perspectives

**DOI:** 10.3389/fbioe.2022.1029403

**Published:** 2022-10-14

**Authors:** Robert Ohlendorf, Andreas Möglich

**Affiliations:** ^1^ Department of Biological Engineering, Massachusetts Institute of Technology, Cambridge, MA, United States; ^2^ Department of Biochemistry, University of Bayreuth, Bayreuth, Germany; ^3^ Bayreuth Center for Biochemistry and Molecular Biology, Universität Bayreuth, Bayreuth, Germany; ^4^ North-Bavarian NMR Center, Universität Bayreuth, Bayreuth, Germany

**Keywords:** biotechnology, gene expression, optogenetics, sensory photoreceptor, signal transduction, synthetic biology

## Abstract

Numerous photoreceptors and genetic circuits emerged over the past two decades and now enable the light-dependent i.e., optogenetic, regulation of gene expression in bacteria. Prompted by light cues in the near-ultraviolet to near-infrared region of the electromagnetic spectrum, gene expression can be up- or downregulated stringently, reversibly, non-invasively, and with precision in space and time. Here, we survey the underlying principles, available options, and prominent examples of optogenetically regulated gene expression in bacteria. While transcription initiation and elongation remain most important for optogenetic intervention, other processes e.g., translation and downstream events, were also rendered light-dependent. The optogenetic control of bacterial expression predominantly employs but three fundamental strategies: light-sensitive two-component systems, oligomerization reactions, and second-messenger signaling. Certain optogenetic circuits moved beyond the proof-of-principle and stood the test of practice. They enable unprecedented applications in three major areas. First, light-dependent expression underpins novel concepts and strategies for enhanced yields in microbial production processes. Second, light-responsive bacteria can be optogenetically stimulated while residing within the bodies of animals, thus prompting the secretion of compounds that grant health benefits to the animal host. Third, optogenetics allows the generation of precisely structured, novel biomaterials. These applications jointly testify to the maturity of the optogenetic approach and serve as blueprints bound to inspire and template innovative use cases of light-regulated gene expression in bacteria. Researchers pursuing these lines can choose from an ever-growing, versatile, and efficient toolkit of optogenetic circuits.

## Introduction

**FIGURE 1:**
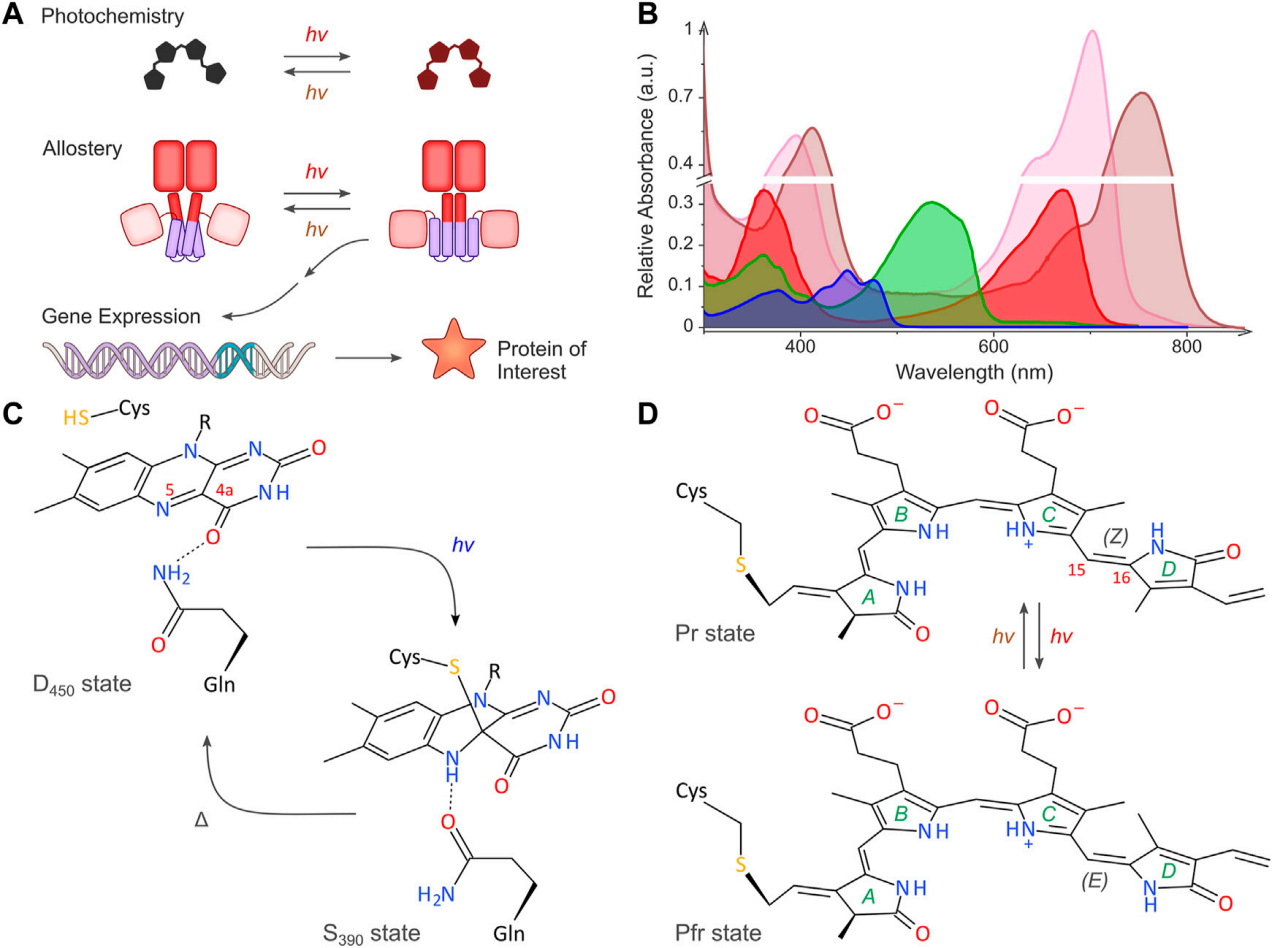
Light-regulated gene expression in bacteria. **(A)** The schematic depicts the basic principles of the optogenetic regulation of bacterial gene expression. Sensory photoreceptors harbor chromophores that undergo photochemical reactions in response to light absorption. The photochemical level is coupled to allosteric transitions within the protein moiety that culminate in altered photoreceptor activity. In turn, the light-induced activity change prompts the bacterial expression of the protein of interest. As illustrated in the scheme, certain photoreceptors are photochromic and can be bidirectionally toggled by two light colors. **(B)** Representative absorbance spectra of select sensory photoreceptors, with light-oxygen-voltage (LOV) and BLUF receptors shown in blue, the paradigm cyanobacteriochrome (CBCR) CcaS in green (Pg state) and red (Pr state), and bacterial phytochromes (BphP) in pink (Pr state) and brown (Pfr state). The spectra are scaled to reflect the individual peak extinction coefficients, i.e., around 12,500 M^−1^ cm^−1^, 30,000 M^−1^ cm^−1^, and 90,000 M^−1^ cm^−1^ for LOV/BLUF, CBCR (Pg), and BphP (Pr), respectively. **(C)** Simplified photochemistry of LOV receptors. Upon absorption of blue light by the dark-adapted state D_450_, a covalent adduct forms between atom C4a of the flavin chromophore and the Sγ atom of a conserved cysteine residue. The resultant protonation of the N5 atom within the signaling state S_390_ is read out by a conserved glutamine residue which undergoes a sidechain flip in response. The signaling state passively decays to the dark-adapted resting state. **(D)** Simplified photochemistry of BphPs. A biliverdin (BV) chromophore (pyrrole rings marked A through D) is covalently linked to a cysteine residue. Within the Pr state, the C15 = C16 double bond adopts the 15*Z* configuration. Upon absorption of red light, this bond isomerizes to the 15*E* state, thus giving rise to the Pfr state. Absorption of far-red light drives the reversion to the Pr state. Conventional BphPs exhibit the Pr form as their dark-adapted state, as opposed to bathyphytochromes which feature the Pfr state in darkness. Although cyanobacterial phytochromes and CBCRs employ the reduced bilin chromophore phycocyanobilin instead of BV, the principal photochemistry hinging on 15*Z*/15*E* isomerization is shared by these receptors, too. CBCRs diversify this fundamental photochemical response to achieve sensitivity to light bands other than red and far-red (Fushimi and Narikawa, 2019).

Light-dependent adaptations of organismal development, behavior, and physiology abound in nature. Well-known examples include vision, photomorphogenesis, phototropism, and phototaxis across diverse organisms (Engelmann, 1883; Butler et al., 1959; Briggs, 2014). Although phenomenologically known early on, many of the mechanistic details of light sensation long awaited elucidation until the molecular identification of the underlying signal circuits. At the molecular stage, light is perceived by sensory photoreceptor proteins which are sensitive to different bands of the near-ultraviolet (near-UV) to near-infrared (NIR) region of the electromagnetic spectrum. Sensory photoreceptors translate photon absorption by their chromophore into changes of their biological activity, for instance enzymatic activity or interaction with other biomacromolecules (Figure 1A). The molecular identification of photoreceptors and an understanding of their inner workings, if often only partial, allowed their deployment in heterologous organisms to modulate by light cellular state and processes, a discipline now known as optogenetics (Deisseroth et al., 2006). Swiftly following their seminal description as light-gated cation-conducting channels (Nagel et al., 2002, Nagel et al., 2003), channelrhodopsins from unicellular algae served to control by light the ion gradient across the plasma membrane and action potentials in mammalian cells (Boyden et al., 2005; Zhang et al., 2007). Particular advantages of this and other optogenetic interventions are the genetic encoding, precise spatiotemporal control, reversibility, and non-invasiveness. Concurrent with these studies or even predating them, two seminal reports harnessed bacterial and plant phytochromes, respectively, for the red-light-dependent control of gene expression in bacteria and yeast (Shimizu-Sato et al., 2002; Levskaya et al., 2005).

By establishing the principal feasibility of optogenetics, these pioneering applications already hinted at a much greater versatility and wider scope of the fundamental approach: evidently, optogenetics is not restricted to neurobiology nor to mammalian cells alone. For one, certain other photoreceptors occurring in nature were of immediate optogenetic utility without any or much modification, arguably best exemplified by the photoactivated adenylyl cyclases from *Euglena gracilis* and *Beggiatoa* sp., respectively (Iseki et al., 2002; Schröder-Lang et al., 2007; Ryu et al., 2010; Stierl et al., 2011). For another, artificial photoreceptors with customized light response were engineered and unlocked additional cellular processes for optogenetics (Shimizu-Sato et al., 2002; Levskaya et al., 2005; Strickland et al., 2008; Möglich et al., 2009; Wu et al., 2009). The latter strategy was to large degree enabled by the discovery of the flavin-binding, blue-light-responsive cryptochrome, LOV (light-oxygen-voltage), and BLUF (sensors of blue light using flavin adenine dinucleotide) photoreceptor classes (Ahmad and Cashmore, 1993; Christie et al., 1998; Gomelsky and Klug, 2002; Iseki et al., 2002). Of key importance and in common with plant and bacterial phytochromes (Butler et al., 1959; Hughes et al., 1997), the LOV and BLUF photoreceptor classes exhibit decidedly modular architecture. In contrast to rhodopsins which are frequently functional as single all-helical transmembrane domains (Rozenberg et al., 2021), in the modular receptors photosensor and effector entities are precisely delineated and can be physically separated. The abundance of naturally occurring, modular (photo)receptors provided blueprints for the construction of artificial photoreceptors *via* recombination of photosensor and effector modules. As a particularly versatile manifestation of this strategy, light-regulated association and dissociation reactions, undergone by many photoreceptors, served to subject manifold target effectors to light control. Owing to the collective efforts of many scientists, a broad set of optogenetic tools is now at hand to govern by light various aspects of cellular physiology and signaling, in both prokaryotes and eukaryotes (Losi et al., 2018; Tang et al., 2021; Govorunova et al., 2022).

Notwithstanding the sheer diversity of optogenetic modalities realized to date, the regulation of gene expression by light remains particularly widespread and versatile (Figure 1A). Although slow in response compared to other optogenetic strategies, light-regulated gene expression provides a general and highly adaptable means of modifying diverse traits of target cells and organisms. Moreover, changes in gene expression elicited by light are generally long-lasting and yield persistent effects, rather than the transient cellular responses of many other optogenetic approaches. Assuming a desired application does not demand utmost temporal resolution as frequently needed in cell biology and the neurosciences, light-regulated gene expression hence often appears as the method of choice for optogenetic control. As reviewed elsewhere (Losi et al., 2018; Tang et al., 2021), several setups for the light-dependent regulation of eukaryotic gene expression emerged in the two decades after the first such system was established for yeast (Shimizu-Sato et al., 2002). Here, we review the current state and recent developments of light-regulated gene expression in prokaryotes. Since the arrival of the initial optogenetic setups for gene expression in bacteria (Levskaya et al., 2005; Möglich et al., 2009; Tabor et al., 2011; Ohlendorf et al., 2012), many more systems were advanced (Baumschlager and Khammash, 2021; Fischer et al., 2022; Hoffman et al., 2022; Lindner and Diepold, 2022; Mazraeh and Di Ventura, 2022; Reshetnikov et al., 2022). In this article, we first recapitulate fundamental aspects of photoreceptors and optogenetics as they pertain to light-regulated gene expression. Next, we move on to the principal strategies currently available for controlling bacterial expression by light. Last, we consider the increasingly numerous and diverse applications in synthetic biology and biotechnology that capitalize on the exquisite spatiotemporal resolution, noninvasiveness, and reversibility afforded by optogenetics.

### Sensory photoreceptors for bacterial optogenetics

Based on chromophore type and the photochemical reactions elicited by light absorption, the sensory photoreceptors identified to date can be grouped into around ten distinct families (Ziegler and Möglich, 2015). Together, these families cover the entire near-ultraviolet to near-infrared section of the electromagnetic spectrum (Figure 1B). Given that the photochemistry, structure, and signaling mechanisms of sensory photoreceptors have been reviewed elsewhere e.g., (Losi et al., 2018; Möglich, 2019; Rozenberg et al., 2021; Tang et al., 2021), the current focus is on salient aspects as they pertain to applications in bacteria. Sensory photoreceptors generally traverse between their dark-adapted (or, resting) and light-adapted (or, signaling) states. Light absorption by the chromophore within the dark-adapted photoreceptor triggers a series of photochemical events, collectively known as the photocycle, and leads to population of the metastable light-adapted state. The initial reaction triggered by photon absorption is generally fast to ensure high quantum efficiency for signal transduction. Several intermediates may occur en route to the signaling state but are short-lived. Given that the lifetime of these intermediates is generally much shorter than the relevant timescales of many cellular signal responses and gene expression in particular, for the present context we only consider the dark-adapted and light-adapted states. With notable exceptions (Ortiz-Guerrero et al., 2011), sensory photoreceptors generally operate reversibly, and the light-adapted state passively, i.e. thermally, reverts to the resting state in the so-called dark-recovery reaction. Certain photoreceptor classes, e.g., bacterial phytochromes and cyanobacteriochromes, are photochromic in that the signaling state can be actively returned to the dark-adapted state *via* absorption of a second photon, usually of different wavelength than the initial photon absorption. The photocycle of photochromic receptors can thus be deliberately abridged to potentially enhance the spatial and temporal precision of optogenetic applications (Ziegler and Möglich, 2015). We note that photochromic reversion to the dark-adapted state also applies to LOV receptors (Losi et al., 2013). Arguably owing to the low efficiency of this process and the requirement for UV radiation, photochromicity in LOV receptors has not been leveraged for bacterial optogenetics to date.

All in all, the strategies for the optogenetic regulation of bacterial expression predominantly harness LOV receptors (Christie et al., 1998; Losi et al., 2018), bacterial and cyanobacterial phytochromes (Chernov et al., 2017; Tang et al., 2021), and cyanobacteriochromes (Rockwell and Lagarias, 2010). By contrast, plant phytochromes and cryptochromes (Shimizu-Sato et al., 2002; Kennedy et al., 2010; Tang et al., 2021), frequently used for optogenetically regulating gene expression in mammalian hosts, have seen scant, if any, use in prokaryotes, arguably due to the size of these receptors and difficulties of functionally expressing them in bacteria. LOV receptors bind flavin-nucleotide cofactors, mostly flavin mononucleotide, to absorb blue light (ca. 420–490 nm) (Christie et al., 2015; Figures 1B,C). The ensuing photocycle features a signaling state characterized by a covalent thioadduct between the flavin chromophore and a conserved cysteine residue of the receptor. The resultant flavin protonation is read out by a conserved glutamine and transduced in form of hydrogen-bonding rearrangements. Intriguingly, neither the cysteine (Yee et al., 2015) nor the glutamine (Dietler et al., 2022) are strictly required for signal transduction; their removal can modulate the absolute light sensitivity and dark recovery of the receptor but generally impairs the fidelity of signaling. With certain exceptions (Rivera-Cancel et al., 2014), bacterial LOV receptors are parallel homodimers that exhibit a range of associated effector modules (Glantz et al., 2016). Other optogenetic tools used in bacteria are based on BLUF photoreceptors (Gomelsky and Klug, 2002). These parallel homodimeric receptors also bind flavin-nucleotide chromophores and thereby sense blue light but differ from LOV receptors in their photochemistry and the structural signal output generated upon photon absorption.

Bacterial phytochromes (BphP) and cyanobacteriochromes (CBCR) are members of the phytochrome superfamily which covalently bind linear tetrapyrrole (bilin) chromophores that undergo light-driven *Z*/*E* isomerization. These receptors are generally photochromic with one light color driving the *Z*→*E* isomerization, and another light color promoting the *E*→*Z* transition. The photosensory core modules (PCM) of BphPs comprise three concatenated domains, denoted PAS, GAF, and PHY (Essen et al., 2008; Yang et al., 2008). A biliverdin (BV) chromophore nestles within the GAF moiety and cycles between its *Z* and *E* isomers that absorb red (ca. 650–700 nm) and far-red light (ca. 700–750 nm), and that are hence referred to as the Pr and Pfr states (Figures 1B,D; Butler et al., 1959). The bilin isomerization couples to a long protein loop, the so-called tongue, emanating from the PHY domain and causes its refolding from a β hairpin in the *Z* isomer to an α helix in the *E* isomer (Anders et al., 2013; Takala et al., 2014). BphPs commonly occur as homodimers, mostly in parallel orientation, and the light-dependent tongue refolding prompts a pivot motion of the two monomeric units. Conventional BphPs assume the *Z* isomer (Pr) as their dark-adapted state, rather than the *E* isomer (Pfr) in the so-called bathyphytochromes. Cyanobacterial phytochromes, exemplified by Cph1 from *Synechocystis* sp. PCC 6803, use the reduced bilin phycocyanobilin (PCB) instead of BV, but resemble BphPs in other regards. Most CBCRs equally use PCB as their light-sensitive pigment but offer compacter architecture in that their PCMs consist of sole GAF domains. Often, CBCR modules are found within serially connected arrays of tandem CBCR and GAF domains (Rockwell et al., 2013). Apart from their smaller footprint, CBCRs garner additional interest because of the diverse photocycles and color sensitivity evidenced in different members of this photoreceptor family (Fushimi and Narikawa, 2019). For instance, the CBCR histidine kinase CcaS from *Synechocystis* sp. PCC 6803, which is frequently used in bacterial optogenetics (Tabor et al., 2011), adopts the *Z*-configured Pg state in darkness that can be converted by green light (ca. 500–600 nm) to the *E*-configured red-light-absorbing Pr state (ca. 600–700 nm) (Hirose et al., 2008; Figure 1B). Irrespective of the enormous color diversity across the CBCR clade, the principal photochemical reaction triggered by light is the photoreversible *Z*↔*E* isomerization of the bilin chromophore around its C15 = C16 double bond (Fushimi and Narikawa, 2019). In nature, CBCR receptors often function as sensor histidine kinases (SHK) but other effectors also occur (Blain-Hartung et al., 2018). Although not yet harnessed for optogenetic actuation in bacteria, a subset of CBCRs incorporate BV rather than PCB, thus resulting in a red-shift of the absorbance spectra in the *Z* and *E* states (Narikawa et al., 2015).

Only identified a decade ago (Ortiz-Guerrero et al., 2011), the CarH-type photoreceptors represent a special case as they feature an irreversible photocycle revolving around 5′-desoxyadenosyl cobalamin (vitamin B_12_) chromophores. Green-light absorption (ca. 500–600 nm) ruptures the metalorganic bond between cobalt and the adenosyl moiety and thereby prompts dissociation of the homotetrameric CarH into monomers (Ortiz-Guerrero et al., 2011; Jost et al., 2015).

A necessary requirement for optogenetic regulation is the *in situ* assembly of the apo-photoreceptor with its chromophore to form the functional holo-receptor. Chromophore uptake and, in case of BphPs and CBCRs, its covalent attachment generally proceed autonomously and do not require additional factors. As described above, LOV and BLUF receptors harbor flavin-nucleotide pigments which are universally present in cells as redox-active cofactors. By contrast, other photoreceptor families rely on chromophores that are specific to certain organisms and may not be present by default in many prokaryotes. The BV and PCB chromophores of BphPs and CBCRs are routinely supplied *via* coexpression of enzymes that generate these bilins from heme. Heme oxygenase (HO), most often HO1 from *Synechocystis* sp. PCC 6803 (Mukougawa et al., 2006; Tabor et al., 2011), mediates the oxidative cleavage of heme to BV. In turn, BV can be reduced to PCB, usually in a single step catalyzed by the ferredoxin-dependent oxidoreductase PcyA, also from *S.* sp. PCC 6803. Although certain microorganisms are capable of synthesizing cobalamin, many prokaryotes are not, and the chromophore thus needs to be supplied exogenously for optogenetic applications of CarH and related photoreceptors. By contrast, the heterologous *in situ* production of this chromophore *via* coexpression of the biosynthetic machinery appears impractical, given that around 30 genes are involved (Fang et al., 2018).

### Light-dependent signal transduction

**FIGURE 2:**
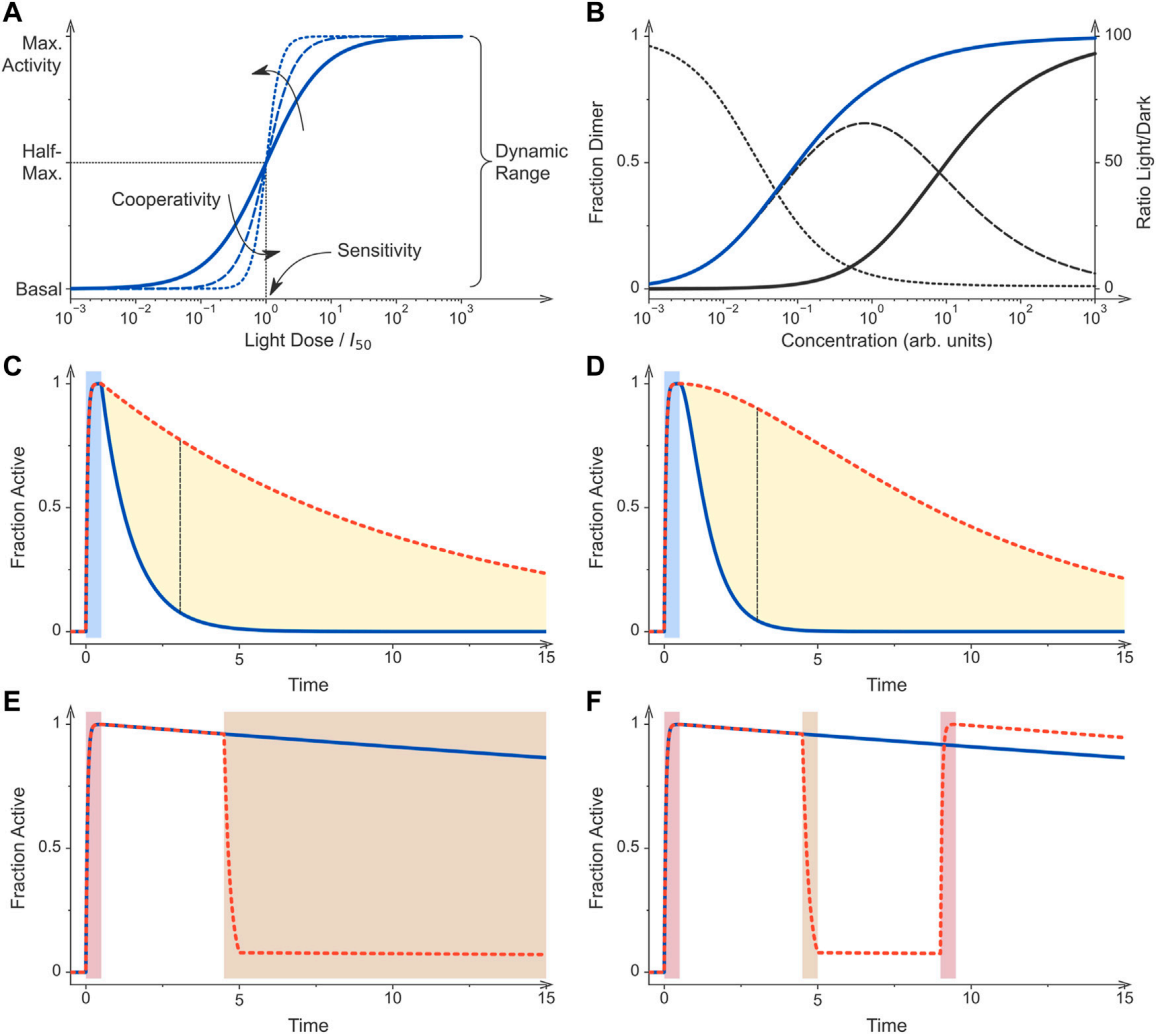
Principles of optogenetic control. **(A)** Sensory photoreceptors and genetic circuits for the light-regulated gene expression in bacteria are characterized by key performance parameters. If all photoreceptor molecules dwell in their low-activity state, the circuit generates a basal output, also denoted as leak activity. Once all photoreceptors are converted to their more active state, maximal activity is generated by the circuit. The ratio of maximal over basal activity is referred to as the dynamic range or regulatory efficiency/factor. Optogenetic circuits differ in their light sensitivity, commonly reported as the light dose required for half-maximal activation, and the cooperativity of their response to illumination. The solid curve shows the response of a non-cooperative optogenetic circuit where *I*_50_ is the light dose at which half-maximal activation occurs. By contrast, the dashed and dotted curves denote circuits that respond to light cooperatively with Hill coefficients of 2 and 4, respectively. **(B)** Many optogenetic tools for controlling bacterial gene expression rely on light-dependent dimerization equilibria. Associating photoreceptors exhibit a lower dissociation constant under blue light (*K*_D_ = 0.1, blue curve) than in darkness (*K*_D_ = 10, black), thus causing their activation profiles to be displaced along the concentration axis. The dashed line denotes the difference of the dimeric receptor fraction in light and darkness for the assumed scenario of a 100-fold changed dissociation constant (left scale). The dotted line plots the ratio of the dimer fractions (right scale). **(C)** Photoreceptors can substantially differ in their dark-recovery kinetics, and for certain classes deliberate residue substitutions near the chromophore modulate these kinetics. After initial stimulation by light (blue bar), a given photoreceptor and hence its activity recover with its intrinsic rate constant. The dashed red curve simulates a receptor that recovers at a tenth of the rate of that for the blue curve. The dashed black line denotes the time point at which the blue and red curves have the maximal difference. **(D)** As in panel C but for a dimeric photoreceptor which is assumed to be active if at least one of its subunits dwells in the light-adapted state. After illumination ceases, the recovery of activity is hence sigmoidal rather than exponential. As a corollary, the maximal difference between the two simulated photoreceptors which differ in their dark-recovery rates by a factor of ten is larger than in scenario C (dashed black line). **(E)** Certain photoreceptor circuits are sensitive to a second stimulus e.g., light of a different color, a chemical inducer, or changes in temperature (Dietler et al., 2021; Romano et al., 2021). Initial photostimulation (red bar) can be counteracted by subsequent application of the second signal (brown bar). **(F)** Photochromic photoreceptors, e.g., phytochromes, can be reversibly and repeatedly toggled between their dark-adapted and light-adapted states by two colors of light. These photoreceptors thus constitute a special case of scenario **(E)**. Following initial photostimulation (red bar), the system can either recover in darkness (blue curve) or be actively returned to the initial state by illumination at desired times (brown bar, red dashed curve). All simulations were conducted with Fit-o-mat (Möglich, 2018).

Before treating in detail the strategies for light-regulated bacterial expression realized to date, we briefly consider general aspects and system characteristics that pertain to optogenetic applications (Ziegler and Möglich, 2015). As introduced above, sensory photoreceptors are in photodynamic equilibrium between their dark-adapted resting state and light-adapted signaling state. The response of a given optogenetic circuit – i.e. in the present context, the expression output generated – will not only depend on the fractional population of these two states but also on the specific activities associated with them (Figure 2A). In case of light-activated circuits, the signaling state has higher specific activity than the resting state, whereas for light-repressed circuits, it is the opposite. Even if all photoreceptors dwell in their low-activity state, optogenetic circuits will generally produce a basal output, also referred to as leakiness. Once all photoreceptors are shifted to their high-activity state, maximal gene-expression output of the circuit will be obtained. The ratio of maximal over basal activity is usually denoted as the dynamic range (or, regulatory efficiency/factor) (Figure 2A), and optogenetic strategies commonly strive to optimize this parameter. The dynamic range is generally improved more effectively by reducing the basal activity rather than by increasing the maximal activity (Ziegler and Möglich, 2015). Another important consideration is how the interconversion between resting and signaling states varies with applied light dose. To the extent it has been studied, many photoreceptors employed for regulating bacterial gene expression follow simple dose-saturation relationships i.e., the degree of receptor activation increases hyperbolically with light dose. The dose at which half-maximal activation occurs determines the light sensitivity of a given system (Figure 2A). Several factors can give rise to cooperativity and thereby cause deviations from the hyperbolic relationship, often incurring sigmoidal or Hill-type relationships. For instance, many, if not most, photoreceptors used in bacterial optogenetics act as homodimers and therefore harbor two light-responsive monomers (Figure 2B). Light-induced conversion of but one of these monomers to the signaling state may impact differently on receptor activity, ranging from no measurable change in output to full effect. As a case in point, the engineered SHK YF1 comprises two LOV entities that can absorb light independently of each other (Möglich et al., 2009). Conversion of just one LOV unit to the signaling state alters receptor activity to the same extent as if both LOV units were converted. Moreover, cellular circuitry that translates photoreceptor activation into gene-expression output may also yield cooperativity (Ziegler and Möglich, 2015). In a similar manner, such circuits could also experience thresholding effects and hence deviate from simple dose-saturation relationships.

The light sensitivity of optogenetic circuits (Figure 2A) is fundamentally linked to how efficiently the underlying sensory photoreceptors absorb light and then undergo productive photochemistry that culminates in population of the signaling state (see Figures 1C,D). Put simply, what are the extinction coefficients and quantum yields for productive photochemistry in different photoreceptors? Although not all relevant photoreceptors have been characterized in this regard, general information for individual photoreceptor classes exists. By virtue of their flavin-nucleotide chromophores, LOV receptors absorb blue light with a maximum around 450 nm where the molar extinction coefficient is around 10,000 to 15,000 M^−1^ cm^−1^ (Figure 1B). The overall quantum yield for formation of the thioadduct signaling state *via* an intermediate triplet state amounts to around 0.3–0.4 for the widely used *Avena sativa* phototropin 1 LOV2 (*As*LOV2) module and to around 0.5 for *Bacillus subtilis* YtvA (Losi et al., 2002; Kennis et al., 2003; Figure 1C). Bacterial phytochromes absorb light in their Pr and Pfr states with maxima at around 700 nm and 750 nm, respectively, and with molar extinction coefficients between ∼70,000–90,000 M^−1^ cm^−1^ (Figure 1B). Not least because the absorption within this spectral band (the so-called Q band) strongly depends on protonation of the bilin chromophore, the molar extinction coefficient at the absorbance maximum varies considerably across individual BphPs. Cyanobacterial phytochromes absorb at shorter wavelength owing to the less extended conjugated π electron system in PCB compared to BV. For both BphPs and Cph1, the quantum yields for productive Pr↔Pfr photoconversion are relatively low, on the order of 0.15–0.2 (Dasgupta et al., 2009; Toh et al., 2010; Figure 1D). CcaS, as the CBCR representative most relevant for bacterial optogenetics, maximally absorbs at 535 nm in its dark-adapted Pg state with a molar extinction coefficient of 27,000 M^−1^ cm^−1^ (Hirose et al., 2010). Once converted to the Pr state, the absorbance maximum shifts to 670 nm, and the extinction coefficient amounts to 30,000 M^−1^ cm^−1^. The quantum yields for driving the *Z*/*E* isomerization and conversion between the two states of CBCRs are about 0.3–0.4 (Slavov et al., 2015) i.e., significantly higher than for BphPs.

Intricately connected to the absorption and photoconversion properties is light delivery *in situ*. The requirements and boundary conditions for light delivery are determined by the given optogenetic application. As detailed below, light-regulated gene expression has been applied to bacteria in diverse contexts, including in dense liquid culture and inside animal hosts. Irrespective of the exact application scenario, light scattering generally scales with the inverse fourth power of wavelength, thus causing shorter wavelengths to be scattered more strongly than long ones. This phenomenon accounts in part for the better tissue penetration of longer wavelengths within the near-UV to NIR region of the electromagnetic spectrum (Weissleder, 2001). Light penetration through biological tissue is additionally limited because of absorption by hemoglobin and other biomolecules. Depending on wavelength, the amount of light that may be delivered per unit time can be narrowly restricted before phototoxicity sets in which may harm living cells and may obscure light-dependent signaling responses. As one workaround, upconverting nanoparticles (UNP) have been used to toggle blue-light-sensitive optogenetic circuits inside the digestive tract of animals (Yang et al., 2020; Cui et al., 2021; Pan et al., 2022). These nanoparticles are activated by NIR light (e.g., 980 nm) which penetrates biological tissue more readily and is less phototoxic than blue light. Multiple absorption events generate a metastable state in the UNPs, out of which a photon of shorter wavelength, e.g., of blue color, is emitted that in turn can trigger the optogenetic system. Notably, the multi-photon excitation of UNPs and subsequent photoluminescence are usually complete within micro- to milliseconds (Gnach and Bednarkiewicz, 2012; Deng and Liu, 2014; Qin et al., 2018) i.e., on a timescale relatively fast compared to the processes targeted by optogenetics, especially gene expression. Hence, the use of UNPs should have no negative impact on the temporal stimulation characteristics. As photons within the NIR range suffer less scattering in biological tissue than those in the visible range, UNPs could well allow spatially more precise stimulation. However, as delivery to the target site *in situ* is required, pertinent approaches are no longer entirely genetically encoded which can be a limitation, depending on use case. A potential remedy are optogenetic circuits that can be triggered by red light such as the Cph8:OmpR setup (Levskaya et al., 2005) or the recently developed pREDusk/pREDawn systems (Multamäki et al., 2022). As a case in point, pREDawn was activated by red light at therapeutically safe intensities through materials with the optical properties of mouse tissue. To potentially enhance sensitivity of a given optogenetic circuit, one might consider modulation of the absolute light sensitivity which is principally governed by the molar extinction coefficient (see Figure 1B) and quantum yield for productive photochemistry. However, modifications to the photoreceptor that would alter this quantum yield are relatively little explored. Moreover, at least for certain photoreceptor classes the experimentally observed photoconversion efficiencies may already approach the physically possible limit, arguably obtained upon ample optimization during evolution. (Evidently, the quantum yield can only be unity or less.) A different and more accessible route towards varying the effective light sensitivity is provided by modulation of the dark-recovery kinetics that determine how fast a photoreceptor thermally reverts from its signaling state to the resting state. In particular for LOV receptors (Christie et al., 2007; Kawano et al., 2013; Pudasaini et al., 2015), but also for BphPs (Yang et al., 2007; Yang et al., 2009), residue exchanges nearby the chromophore are known to decelerate or accelerate the dark recovery. Optogenetic applications, especially those involving light-regulated gene expression, are frequently performed under photostationary conditions, and the underlying photoreceptors may undergo repeated cycles of photoactivation and subsequent recovery. The fraction of the receptor in its signaling state is hence governed by the effective light sensitivity i.e., the balance between the velocities of the light-driven activation and the passive recovery (Ziegler and Möglich, 2015). The targeted variation of recovery kinetics thus provides a handle to substantially modulate the sensitivity of optogenetic circuits at photostationary state (Ziegler and Möglich, 2015; Hennemann et al., 2018). However, caution must be exerted, as certain residue exchanges were found to not only modulate the recovery kinetics but to also negatively affect signal transduction e.g., (Diensthuber et al., 2014; Dietler et al., 2022).

The reactions leading to population and depletion, respectively, of the signaling state also contribute to the temporal resolution that can be achieved for a specific optogenetic application. Since the photochemical reactions playing out in photoreceptors after light absorption are fast compared to downstream responses, they are generally not limiting for the turn-on kinetics with which an optogenetic circuit can be triggered. Rather, these kinetics are more often limited by light delivery *in situ*, see above, and slower subsequent reaction steps. The latter consideration certainly holds true for light-regulated gene-expression systems which in most cases realize regulation at the transcriptional level, see control points for optogenetic regulation below. Although the experimental data on this aspect are sparse, several optogenetic setups for light-regulated expression in bacteria exhibited significant changes in expression levels within around half an hour after light exposure, with the response taking several hours to manifest to full degree (Ohlendorf et al., 2012; Olson et al., 2014, 2017; Ramakrishnan and Tabor, 2016; Ong and Tabor, 2018; Multamäki et al., 2022; Ranzani et al., 2022). The dark-recovery kinetics greatly differ across photoreceptor families and their individual members. For example, plant phototropin LOV domains, exemplified by the widely deployed *As*LOV2, recover to the resting state with time constants around 100 s or less (Kottke et al., 2003) which contrasts with the much slower kinetics on the order of thousands of seconds evidenced in bacterial and fungal LOV domains (Losi et al., 2002; Zoltowski et al., 2007; Möglich et al., 2009; Conrad et al., 2013; Weber et al., 2019). The dark recovery in BphPs is commonly multiphasic and progresses over several thousands of seconds (Multamäki et al., 2021). Within the CBCR family, the dark-reversion kinetics greatly vary and can take between seconds and several hours to complete (Chen et al., 2012; Rockwell et al., 2012; Fushimi et al., 2017). While the popular CcaS receptor recovers to its resting state exceedingly slowly, the widely used Cph8 does so in around 5 minutes (Olson et al., 2017). As discussed above, at least for certain photoreceptor families, residue exchanges near the chromophore have been identified which greatly change the recovery kinetics (Figure 2C). Moreover, the dark recovery is often associated with sizeable activation energies which renders its kinetics strongly dependent on temperature. Whereas the dark recovery usually follows single- or multiexponential courses, the reversion of the biological output upon withdrawal of light need not necessarily track this time course. As experimentally shown and discussed in Figure 2D, oligomeric photoreceptors may react to light cooperatively. For instance, blue light converts the homodimeric YF1 receptor from kinase to phosphatase activity (Möglich et al., 2009). Returned to darkness, the original kinase activity recovers in sigmoidal manner as it requires the reversion of both its LOV monomers to their resting states. Photochromic receptors, such as the particularly widely used SHKs CcaS (Hirose et al., 2008) and Cph8 (Levskaya et al., 2005), enable the active reversion to the resting state by absorption of a second photon of different color (Figures 1A, 2E). Thereby, the reversion reaction can be much accelerated to the extent that it is only limited by light delivery, as discussed above for the forward reaction. The ability to fast and photoreversibly toggle between two activity states provides a decisive advantage for many use cases, not least for the all-optical feedback control of bioproduction processes (Milias-Argeitis et al., 2016; Chait et al., 2017; Steel et al., 2020; Kumar and Khammash, 2022). Pertinent applications have been realized for the green-/red-light-responsive CcaRS two-component system. By continuously monitoring the output of the optogenetic system e.g., cell density or reporter fluorescence, the CcaS receptor can be clamped at desired ratios of its Pg and Pr states, and the system output can thereby be controlled with precision in time exceeding that for non-photochromic receptors. Although not studied in detail, sensory photoreceptors exhibit low photofatigue and can be excited multiple times with little, if any, loss of responsiveness (Figure 2F). Principally, at some point photodamage will accumulate, but for current applications to optogenetically control bacterial expression, there is little indication that photofatigue could be limiting.

Taken together, the different photoreceptor families each offer traits that can be advantageous or limiting, depending on the application scenario. The photochromic, bidirectional toggling of optogenetic circuits is clearly beneficial for many situations. Moreover, the systems based on CBCRs and BphPs generally absorb at longer wavelengths than the widespread blue-light-sensitive LOV receptors, which may prove advantageous when light delivery is limiting. At the same time, CBCRs and especially BphPs absorb across substantial portions of the near-UV to NIR spectrum which may complicate multiplexed applications with other photoreceptors and fluorescent reporters. For instance, the PCM of the *Deinococcus radiodurans* BphP (*Dr*PCM) can not only be activated by red light but also by blue light, owing to its absorption in the Soret band around 400 nm (see Figure 1A) (Gasser et al., 2014). It may furthermore be challenging to fully interconvert between the two spectral states because the absorbance spectra of the two metastable states in photochromic receptors generally overlap (see Figure 1A). Moreover, the quantum yields for the light-driven, forward and reverse reactions may substantially differ. As a case in point, the *Dr*PCM, often used in optogenetics (Tang et al., 2021; Lehtinen et al., 2022), exhibited sluggish Pfr→Pr reversion when illuminated with NIR light (Gasser et al., 2014; Etzl et al., 2018; Stabel et al., 2019). The *in situ* supply of the chromophores for CBCRs and BphPs is usually not limiting as bilin synthesis can be readily achieved *via* coexpression of HO (and PcyA) and is therefore not restricting most bacterial applications. In summary, there is no clear-cut case for generally preferring one photoreceptor class over another. Rather, the availability of several classes with different light sensitivity can be considered an advantage as it enables multiplexed applications of light-regulated bacterial expression, see below (Tabor et al., 2011; Fernandez-Rodriguez et al., 2017; Olson et al., 2017; Multamäki et al., 2022). Lastly, several strategies that proved successful for the design of optogenetic circuits can be often extended to other photoreceptor classes. This is particularly true for circuits that rely on light-controlled oligomerization reactions and, to lesser extent, two-component systems, both of which we discuss in the next section.

### Allosteric mechanisms in light-dependent signal transduction

**FIGURE 3:**
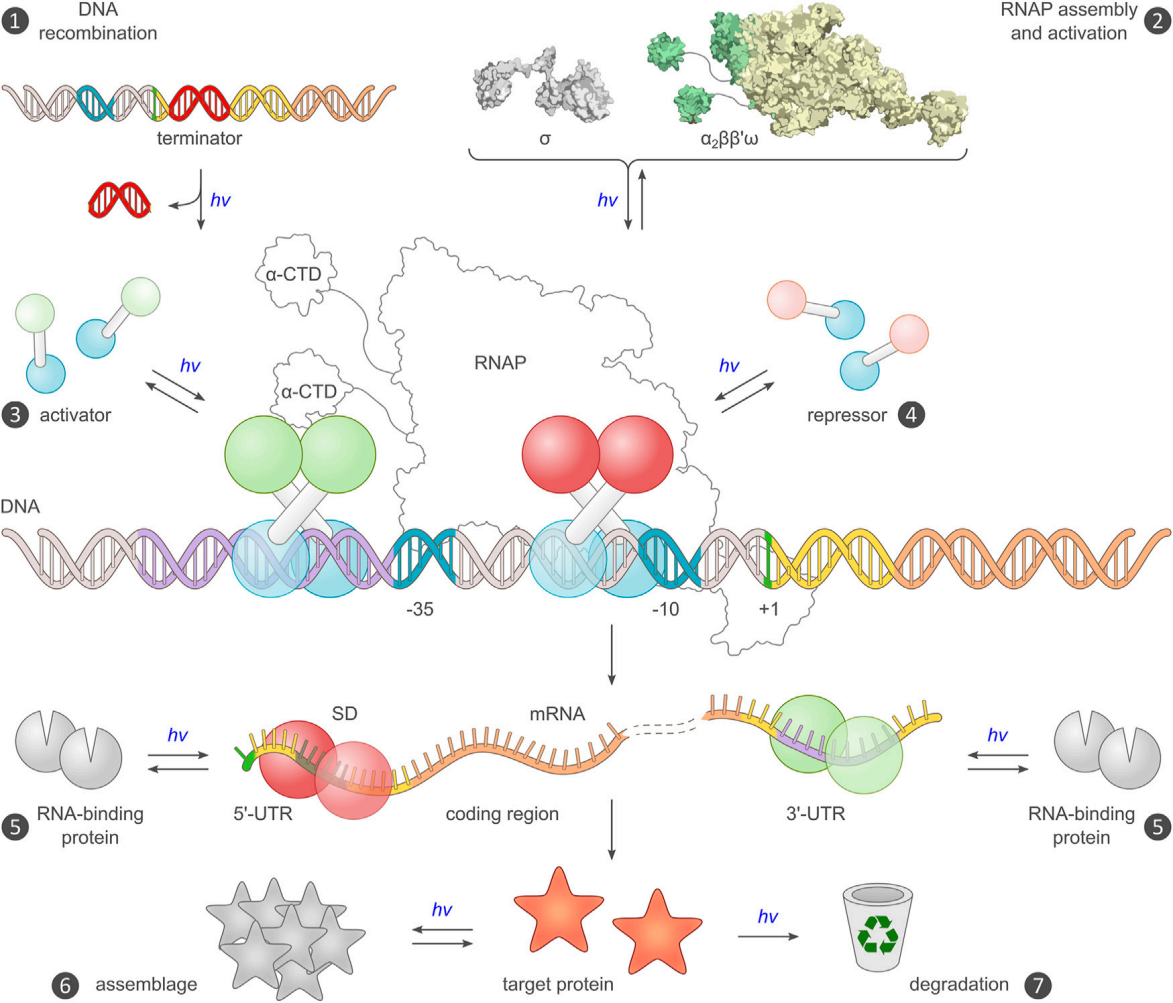
Existing and potential toeholds for the optogenetic control of bacterial gene expression. Expression requires a DNA template encoding an intact transcriptional unit, and the provision of this unit can be modulated by recombination events (mechanism ❶) (Sheets et al., 2020). Alternatively, the assembly of a functional RNA polymerase (RNAP) holoenzyme can be modulated (❷), for example by controlling the association of the endogenous core polymerase (α_2_ββ′) with its σ factor, or by reconstitution of a split viral polymerase (Baumschlager et al., 2017; Han et al., 2017). The main checkpoint for transcription is initiation, which is promoted by activator proteins, often *via* productive interactions with the C-terminal domain of the RNAP α subunits (α-CTD), and hindered by repressors (❸, ❹). Prokaryotic activators and repressors are usually homodimeric, and their activity can hence be governed by controlling their assembly by light, either directly e.g., in case of EL222 (Nash et al., 2011), or indirectly in case of the widespread two-component systems (Levskaya et al., 2005; Möglich et al., 2009; Tabor et al., 2011). At the mRNA level, expression control can be accomplished by directing RNA-binding proteins e.g., PAL (Weber et al., 2019), to the 5′- and 3′-untranslated regions (UTR) of the transcript (❺). Binding to the 5′-UTR may mask the ribosome-binding site (or, Shine-Dalgarno [SD] sequence) and thereby interfere with translation initiation (Ranzani et al., 2022). Alternatively, binding to the 3′-UTR could be harnessed for regulating intracellular RNA stability (Tang and Guest, 1999). Post expression, target proteins can be controlled by binding to other proteins or sequestration into protein clusters, which may incur activity modulation (❻), or by governing their intracellular lifetime and eventual degradation (❼). Beyond the generic strategies in the schematic, numerous proteins were also subjected to light-dependent allosteric control *via* direct fusion with a photoreceptor unit.

Recent years have witnessed the advent of various setups for the light-dependent control of bacterial gene expression (Table 1; Figure 3). Despite this welcome diversity, the vast majority of approaches employ one of merely three principal mechanisms to achieve light sensitivity: i. two-component systems; ii. oligomerization; iii. second messengers.

**TABLE 1:** Strategies for light-regulated gene expression in bacteria.

Name	Photoreceptor mechanism	Target process and mechanism	Dynamic range^a^	Chromophore/color sensitivity	References
OptoCreVvd (split Cre-*Nc*VVD/Magnets)	split protein, dimerization	recombinase reconstitution	+12 × (estimated from figure)^b^	FMN/blue	Sheets et al. (2020)
OptoFlpVvd (split Flp-*Nc*VVD/Magnets)	split protein, dimerization	recombinase reconstitution	+6 × (estimated from figure)	FMN/blue	Sheets et al. (2020)
OptoT7 (split T7, Magnets)	split protein, dimerization	polymerase reconstitution	+332 ×	FMN/blue	Baumschlager et al. (2017)
split T7-*Nc*VVD/Magnets	split protein, dimerization, allostery	polymerase reconstitution and activity	+50–100 × (estimated from figure)	FMN/blue	Han et al. (2017)
split T7-*At*PhyB:PIF3	split protein, dimerization, intein processing	polymerase reconstitution	+5 × (lycopene production)	PCB/red and far-red	Raghavan et al. (2020)
Cph8:OmpR	TCS	transcriptional activation	−9 ×	PCB/red and far-red	Levskaya et al. (2005)
			−72 ×		Schmidl et al. (2014)
pDusk (YF1:FixJ)	TCS	transcriptional activation (plus inverter)	−15 ×	FMN/blue	Ohlendorf et al. (2012)
pDawn	+ λ cI repressor		+460 ×		
OptoLAC (YF1:FixJ)	TCS, λ cI and lacI repressors	transcriptional activation (plus inverter)	−61 ×	FMN/blue	Lalwani et al. (2021a)
YGS24:GacA	TCS	transcriptional activation	+10 ×	FMN/blue	Cheng et al. (2021)
CcaS:CcaR	TCS	transcriptional activation	+6 ×	PCB/red and green	Tabor et al. (2011)
			+117 ×		Schmidl et al. (2014)
			+593 ×		Ong and Tabor (2018)
UirS:UirR	TCS	transcriptional activation	+6 ×	PCB/UV-violet and green	Ramakrishnan and Tabor (2016)
pREDusk (*Dr*F1:FixJ)	TCS	transcriptional activation (plus inverter)	−200 ×	BV/red and far-red	Multamäki et al. (2022)
pREDawn	+ λ cI repressor		+70 ×		
BphP1:PpsR2	heterodimerization, allostery	transcriptional repression	+2.5 ×	BV/red and far-red	Ong et al. (2018)
LOV-TAP (*As*LOV2-TrpR)	allostery	transcriptional repression	n.d.	FMN/blue	Strickland et al. (2008), Strickland et al. (2010)
LightOff LEVI (LexA-*Nc*VVD)	dimerization	transcriptional repression (plus inverter)	−10,000 ×	FMN/blue	Chen et al. (2016)
LEVIon	+ λ cI repressor		+1,000 ×		
LexRO (LexA_408_-*Rs*LOV)	dimer dissociation	transcriptional repression	+500 ×	FMN/blue	Li et al. (2020)
iLight (LexA_408_-*Is*PCM)	dimer-tetramer association	transcriptional repression	−115 ×	BV/red and far-red	Kaberniuk et al. (2021)
pLITR (TetR-*Rs*LOV)	dimer dissociation	transcriptional repression	+14 ×	FMN/blue	Dietler et al. (2021)
pLATR (TetR-*Nc*VVD, TetR-*Pt*aur)	dimerization		−75 ×		
TRU (TetR-*Nc*VVD)	split protein, dimerization	transcriptional repression (plus inverter)	−13 ×	FMN/blue	Komera et al. (2022)
TAU	+ lacI repressor		+5 ×		
CarH	allostery, tetramerization	transcriptional repression	+10 × (LacZ reporter in *Myxococcus xanthus*)	B_12_/green	Ortiz-Guerrero et al. (2011)
pBLind	dimerization, allostery	transcriptional activation	+5 ×	FMN/blue	Jayaraman et al. (2016)
pBLrep		transcriptional repression	−3 ×		Ding et al. (2020)
BLAT (EL222)			+24 ×		
BLRT (EL222)			−53 ×		
pEL EL222			−5 ×		Camsund et al. (2021)
BLADE (*Nc*VVD-AraC, AraC-*Vf*LOV)	dimerization	transcriptional activation	+15 ×	FMN/blue	Romano et al. (2021)
bPAC	cAMP second messenger + CAP	transcriptional activation	+300 × (cAMP production)	FAD/blue	Ryu et al. (2010), Stierl et al. (2011)
IlaC	cAMP second messenger + CAP	transcriptional activation	+6 × (cAMP production)	BV/red and far-red	Ryu et al. (2014)
PaaC			+4 ×		Etzl et al. (2018)
*Dd*PAC			+7 ×		Stüven et al. (2019)
mPAC	cAMP second messenger + CAP	transcriptional activation	+30 × (cAMP production)	FMN/blue	Raffelberg et al. (2013)
cPAC	cAMP second messenger + CAP	transcriptional activation	+3 × (cAMP production)	PCB/blue and green	Blain-Hartung et al. (2018)
*An*PixJg2-AC	cAMP second messenger + CAP	transcriptional activation	+2–3 × (cAMP production)	PCB/red and green	Fushimi et al. (2017)
PaaG	cGMP second messenger	transcriptional activation	+14 × (cGMP production)	BV/red and far-red	Etzl et al. (2018)
BphS	c-di-GMP second messenger + MrkH	transcriptional activation	+40 × (LacZ)	BV/red and far-red	Ryu and Gomelsky (2014)
pCrepusculo (*Nm*PAL)	allostery	translational repression	−10 ×	FMN/blue	Weber et al. (2019), Ranzani et al. (2022)
pAurora	+ λ cI repressor		+67 ×		
LicV (LicT-*Nc*VVD)	dimerization	transcriptional termination	+17 ×	FMN/blue	Liu et al. (2022)
PRU (TEV protease-*Nc*VVD)	split protein, dimerization + cleavable LAA tag	protein degradation	−12 ×	FMN/blue	Komera et al. (2022)
PAU			+4 ×		

^a^ Unless stated otherwise, the listed dynamic ranges are based on the expression of fluorescent reporter genes.

^b^ Positive factors denote an induction of gene expression by light, whereas negative factors signify a reduction of expression under light.

#### Light-responsive two-component systems

**FIGURE 4:**
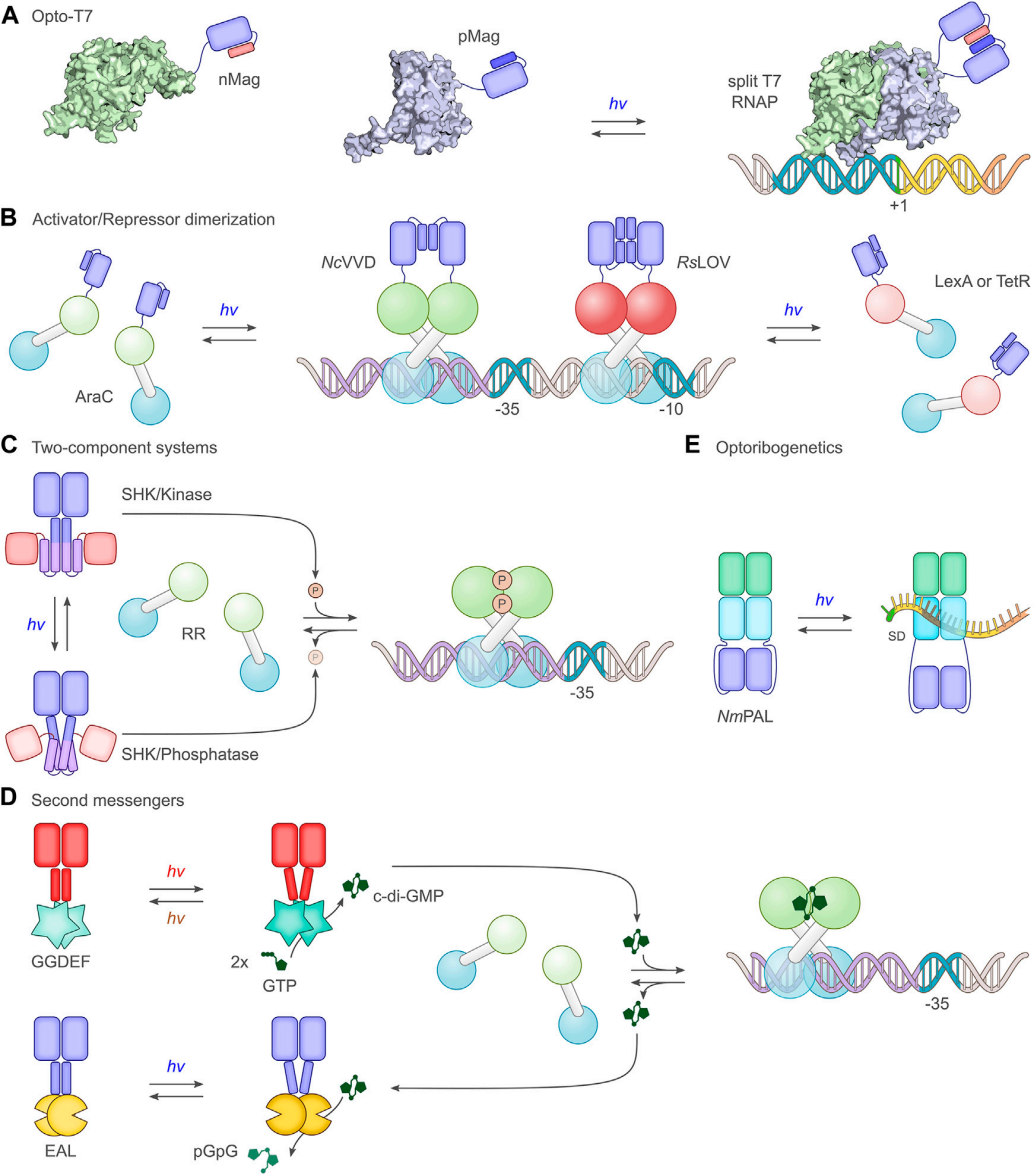
Select principal strategies for the light-dependent control of gene expression. **(A)** The phage-derived T7 polymerase can be dissected into two parts which in separation have little activity. By fusing the polymerase fragments to photoassociating LOV domains, such as the Magnets (Kawano et al., 2015), T7 can be reconstituted under blue light, and transcription ensues (Baumschlager et al., 2017; Han et al., 2017). **(B)** A group of strategies exploit the homodimeric nature of bacterial activator and repressor proteins. *Via* truncation, dimerization can be impaired, thus rendering monomers with little DNA affinity, let alone regulatory effects. As in strategy A, linkage to photoassociating LOV modules, such as *N. crassa* Vivid (*Nc*VVD) (Zoltowski and Crane, 2008), allows dimerization and activity to be regained upon blue-light exposure. For instance, the widely used AraC transcriptional activator was subjected to light control thus (Romano et al., 2021). In a similar vein, repressor proteins such as LexA (Li et al., 2020) or TetR (Dietler et al., 2021) can be monomerized through truncation and linked to the photodissociating *R. sphaeroides* LOV domain (*Rs*LOV) (Conrad et al., 2013). Light exposure leads to a dissociation of the LOV-linked repressor and hence to transcriptional activation. **(C)** A large group of studies (Levskaya et al., 2005; Tabor et al., 2011; Ohlendorf et al., 2012) employ two-component systems, consisting in their canonical form of a sensor histidine kinase (SHK) and a response regulator (RR). Depending on illumination, photoresponsive SHKs adopt kinase-active or phosphatase-active states (Möglich, 2019), thus promoting RR phosphorylation and dephosphorylation, respectively. Once phosphorylated, the RR serves as a transcriptional activator at target promoters. As depicted in the scheme, certain SHKs act as net kinases in their dark-adapted states before being converted to their phosphatase-active states by light (Levskaya et al., 2005; Möglich et al., 2009), while other SHKs are phosphatase-active in darkness and become kinase-active under light (Tabor et al., 2011). **(D)** Several optogenetic strategies are based on cyclic-nucleotide second messengers, in particular 3′, 5′-cyclic adenosine monophosphate and, as shown in the scheme, 3′, 5′-cyclic-diguanylate (c-di-GMP). Activated by light, diguanylate cyclases (GGDEF) catalyze the formation of c-di-GMP which binds and thereby activates specific transcriptional activators (Ryu and Gomelsky, 2014). The hydrolysis of c-di-GMP is mediated by EAL phosphodiesterases, certain of which are also responsive to light (Huang et al., 2018). **(E)** The LOV receptor *Nm*PAL from *N. multipartita* binds specific RNA sequences upon blue-light activation. Once embedded adjacent to the Shine-Dalgarno sequence (SD) of target mRNAs, light-induced PAL binding to these sequences interferes with ribosome binding and reduces expression (Weber et al., 2019; Ranzani et al., 2022).

In their canonical form (Buschiazzo and Trajtenberg, 2019; Möglich, 2019; Lazar and Tabor, 2021), two-component systems (TCS) comprise a sensor histidine kinase (SHK) and a response regulator (RR) (Figure 4C). While SHKs often span the plasma membrane, light-sensitive variants, based on LOV, CBCR, or BphP sensor modules, are soluble proteins. To date, the three most widely used light-responsive TCSs are based on the CcaS CBCR (Tabor et al., 2011; Ong and Tabor, 2018), the cyanobacterial Cph8 (Levskaya et al., 2005; Schmidl et al., 2014), and the LOV receptor YF1 (Möglich et al., 2009; Ohlendorf et al., 2012). SHKs adopt two principal functional states that exert kinase and phosphatase activities, respectively, towards their cognate RRs (Russo and Silhavy, 1993; Möglich et al., 2009; Möglich, 2019). In their kinase-active state, SHKs autophosphorylate at a conserved histidine residue and transfer phosphoryl groups to a conserved aspartate within the RR. When active as a phosphatase, the SHK mediates the hydrolysis of the phosphate anhydride in the phosphorylated RR. The biological output, most often DNA binding and transcriptional activation, is primarily determined by the degree of RR phosphorylation and hence by the net balance between the elementary kinase and phosphatase activities. Although the tug of war between the opposing kinase and phosphatase reactions can incur futile ATP hydrolysis, it also provides the basis for highly stringent and steep signaling responses i.e., low basal activity and high dynamic range. The recognition between SHK and RR is highly specific, and multiple coexisting TCS inside the bacterial cell are well insulated from each other to prevent undesired crosstalk (Skerker et al., 2008). However, heterologous applications of TCSs e.g., for light-regulated gene expression, may potentially suffer from inadvertent crosstalk with endogenous SHKs and RRs, although these aspects are rarely investigated in detail. Similarly, the SHK and its cognate RR may also be subject to non-enzymatic phosphorylation by reactive phosphate species, such as acetyl phosphate. Not least because of these considerations it becomes clear that usually both the kinase and phosphatase modes are important for (heterologous) applications of TCSs. At the molecular level, the transition between kinase and phosphatase modes is mediated by structural rearrangements within the histidine-kinase effector module (Trajtenberg et al., 2016; Möglich, 2019). Signals emanating from the (light-sensitive) SHK sensor module travel to the effector through α-helical coiled coils which exhibit a seven-residue, i.e. heptad, periodicity in their structure. Targeted length modification of the coiled-coil linker thus provides a handle for reprogramming the signal response of SHKs (Möglich et al., 2009; Nakajima et al., 2016b; Ohlendorf et al., 2016). For instance, elongation of said linker converted YF1 from a blue-light-repressed net histidine kinase to a light-activated one (Ohlendorf et al., 2016). Alternatively, certain point mutations in the LOV photosensor sufficed for inverting the response of YF1 to light (Gleichmann et al., 2013; Diensthuber et al., 2014).

#### Light-dependent oligomerization reactions

Many processes in biology rely on protein oligomerization and therefore lend themselves to optogenetic regulation *via* light-dependent association and dissociation reactions. This notion is duly reflected in the manifold setups for light-regulated bacterial gene expression that harness photoreceptor pairs which associate or dissociate under light e.g., (Chen et al., 2016; Li et al., 2020; Dietler et al., 2021; Romano et al., 2021). These approaches have in common that the intrinsic oligomeric state, in most cases dimeric, of a target effector e.g., a transcriptional activator, is disrupted, usually by protein truncation (Figures 4A,B). The truncated protein ideally has little remaining dimerization capability, and its biological activity is thus turned off. Ligation to photoassociating or photodissociating photoreceptor pairs can restore the dimeric state in dependence of light and thus regain biological activity. The application scope of light-dependent association reactions extends to split proteins which are severed into two parts with low mutual affinity (Baumschlager et al., 2017; Sheets et al., 2020) (Figure 4A). Again, light-dependent heterodimerization of the split fragments restores biological activity. A number of protein modules, mostly from the LOV receptor family, serve as light-activated dimerization modules for the optogenetic control of prokaryotic expression. As the most extensively used module, the short-LOV protein Vivid from *Neurospora crassa* (*Nc*VVD) associates under blue light into a homodimer (Zoltowski and Crane, 2008; Vaidya et al., 2011). By modifying residues at the dimer interface, the so-called Magnet pairs were devised which assemble into a heterodimer under blue light while the homodimer affinity of each Magnet component alone is low (Kawano et al., 2015). Similar to *Nc*VVD, the LOV domains *Vf*LOV and *Pt*LOV from aureochromes of stramenopile algae (e.g., *Vaucheria frigida*) and diatoms (e.g., *Phaeodactylum tricornutum*) associate into homodimers upon blue-light absorption (Takahashi et al., 2007; Pfeifer et al., 2010). The short-LOV receptor from *Rhodobacter sphaeroides* (*Rs*LOV) exhibits the opposite response to photon absorption and adopts homodimeric and monomeric states in darkness and under blue light, respectively (Conrad et al., 2013). The optogenetic output generated by systems employing light-regulated association is fundamentally determined by the law of mass action for the oligomerization equilibria in darkness and under light (Figure 2B). As illustrated for a homodimeric photoreceptor, the dimeric fraction of the receptor most strongly varies with illumination at a concentration between the dissociation constants for the dark- and light-adapted states. By contrast, the ratio of the dimeric fractions under light and in darkness monotonically decreases with the receptor concentration. Depending on the value of the dissociation constants, only certain concentration windows may support robust light-induced signaling responses (Figure 2B). Precise data on the light-dependent dissociation constants of photoreceptors are lamentably sparse but values around 10 µM and 0.5 µM were reported for the light-adapted states of *Nc*VVD and *Vf*LOV, respectively (Zoltowski and Crane, 2008; Nakatani and Hisatomi, 2015), with the affinity in the dark-adapted state too weak to be reliably determined. The dissociation constant for the dark-adapted *Rs*LOV homodimer amounted to 40 µM (Dietler et al., 2021), whereas the interaction in the light-adapted state was too weak to be measured. Certain LOV receptors, including *Rs*LOV, are intrinsically temperature-sensitive and may exhibit reduced light responsiveness at elevated temperatures (Dietler et al., 2021; Benman et al., 2022). Numerous optogenetic applications in mammalian cells employ plant cryptochrome 2 (Kennedy et al., 2010; Bugaj et al., 2013), the iLID system (Guntas et al., 2015), the UV-responsive UVR8 (Chen et al., 2013), or plant phytochromes (Levskaya et al., 2009; Golonka et al., 2019) to effect light-dependent oligomerization reactions. To date, these and yet other dimerization systems (Klewer and Wu, 2019) have not seen much use in prokaryotes, likely due to the availability of the above LOV-based, well-performing systems and, at least in certain cases, challenges in the heterologous expression of plant photoreceptors.

#### Light-controlled second-messenger signaling

A third group of approaches for light-regulated gene expression in bacteria harness the production of second messengers which, among other responses, can activate transcription (Figure 4D). The two most prominent types within this group are based on either 3′,5′-cyclic adenosine monophosphate (cAMP) or 3′,5′-cyclic diguanylate (c-di-GMP). Several photo-activated adenylyl cyclases (PAC), which catalyze cAMP production upon light stimulation, were identified in nature or constructed by protein engineering. The most widely used PAC is the one from *Beggiatoa* sp. (Ryu et al., 2010; Stierl et al., 2011) that encompasses a BLUF photosensor and upregulates cAMP synthesis under blue light by several hundred-fold. Other PACs bear BphP and CBCR photosensor modules, thereby unlocking longer wavelengths for the optogenetic regulation of cAMP metabolism, but generally suffer from comparatively low dynamic ranges (Ryu and Gomelsky, 2014; Fushimi et al., 2017; Blain-Hartung et al., 2018; Etzl et al., 2018; Stüven et al., 2019). By contrast, c-di-GMP cyclases linked to BphP PCMs can exhibit exquisite dynamic ranges for regulation by red light (Ryu and Gomelsky, 2014; Gourinchas et al., 2019). Signal transduction in these homodimeric photoactivated cAMP and c-di-GMP cyclases employs light-dependent rearrangements within a helical bundle or coiled coil connecting the photosensor and effector moieties (Gourinchas et al., 2017). The light-modulated levels of the cyclic nucleotides are linked to gene expression *via* transcription factors that are sensitive to these second messengers, see control points for optogenetic regulation below.

#### Comparison of allosteric strategies

Light-sensitive TCSs currently dominate the optogenetic control of bacterial expression. This predominance may in part reflect the comparatively early availability of the YF1, Cph8, and CcaS SHKs which afforded stringent light responses (Levskaya et al., 2005; Möglich et al., 2009; Tabor et al., 2011; Ohlendorf et al., 2012). As another potential reason, TCSs can mediate particularly stringent and pronounced signal responses, owing to the dual kinase and phosphatase activities of their SHKs, see above (Russo and Silhavy, 1993; Möglich, 2019). This inherent property of most SHKs almost certainly accounts for the predominance of TCSs in bacterial signal transduction and may also explain their success in bacterial optogenetics. As implied by their name, TCSs commonly require at least two polypeptide components, namely the SHK and the RR, plus potentially additional accessory components e.g., for chromophore production. This contrasts with systems based on light-dependent homodimerization which experience increased use and are mostly realized as single components (Motta-Mena et al., 2014; Chen et al., 2016; Dietler et al., 2021; Romano et al., 2021). The simpler architecture of these setups appears immediately attractive, not least because it entails a smaller genetic footprint, i.e. the total size of the gene(s) encoding the optogenetic circuit. However, it is unclear to what extent the simpler buildup plays out in practice and grants relevant benefits for current optogenetic applications in bacteria. One might implicitly assume that single-component systems provide more stringent and robust light responses, but this sentiment is not supported by the available data. In fact, thresholding and saturation effects aside (see above), the response of any circuit that banks on oligomerization, be it light-responsive or not, must evidently scale with protein concentration (Figure 2B). Variations in protein concentration could for instance arise from expression differences between cells, even within a monoclonal population (Ziegler and Möglich, 2015). As far as it has been studied, this principal aspect is borne out by experiment (Romano et al., 2021). Although the performance of TCSs will also depend on the amounts of the SHK and RR components (Schmidl et al., 2014), it is potentially less affected by concentration variation, given that both the elementary kinase and phosphatase reactions depend on the SHK and RR concentrations in the same order. Moreover, the binding mode of the RR to the SHK is strikingly similar in the kinase and phosphatase states (Trajtenberg et al., 2016; Möglich, 2019). In line with this observation, the affinity of the *D. radiodurans* BphP (*Dr*BphP) for its RR is little affected by illumination with red and far-red light (Multamäki et al., 2021).

## Control points for optogenetic regulation of bacterial expression

After covering fundamentals of optogenetics in bacteria, we now turn to concrete strategies which subjected bacterial gene expression to light control. As stipulated by the central dogma of molecular biology (Crick, 1958), the genetic information laid down in the DNA is transcribed into RNA before being translated into protein. Optogenetics can principally exert control at different stages of this event chain, as borne out by diverse strategies realized to date for light-regulated expression in *Escherichia coli* and other bacteria (Table 1). Although the most important and most frequently controlled step is transcription initiation, other stages were also controlled optogenetically (Figure 3). When applying optogenetics to the control of bacterial gene expression, a key consideration is how efficient the regulation by light will eventually be. Put another way, what is the dynamic range for regulation by light in the diverse optogenetic strategies at hand (Figure 2A)? Although this question is phrased easily, it is very challenging to answer conclusively. The original reports on the development of the respective optogenetic tools commonly assessed the dynamic range of regulation using reporter genes, in most cases fluorescent proteins, but also β-galactosidase (LacZ). However, the individual studies greatly differ in terms of reporter identity, experimental conditions, data evaluation, and background correction, all of which impact on the attainable regulatory efficiency. Even though a systematic side-by-side comparison between different optogenetic strategies for the control of bacterial gene expression seems principally desirable, no unbiased analyses have yet been undertaken to this effect. Such endeavors would in any case be fraught with substantial challenges, not least that the experimental setting selected for comparison may inadvertently favor one or another of the strategies. Against this backdrop, in the following we refrain from a quantitative comparison of the various optogenetic systems and refer to the dynamic ranges of light regulation provided in the original reports or, where applicable, their later improvements (see Table 1). When appraising the regulatory efficiency for a given setup, one should also consider at which stage of the gene expression process the setup acts. By and large, the response to signal, light or otherwise, is often more pronounced in circuits that operate at the transcriptional level compared to, for instance, the translational level. Beyond dynamic range and basal activity, other aspects are also important for practical application, e.g., sensitivity, light color, phototoxicity, cytotoxicity, and response kinetics, as outlined above. Potential crosstalk with the endogenous bacterial signaling circuits is relevant as well but rarely probed in detail; hence, little concrete data are available on that score. Given that certain optogenetic tools are based on common *E. coli* transcription factors (Levskaya et al., 2005; Chen et al., 2016; Romano et al., 2021), interference with the intrinsic signaling pathways and inadvertent activation of endogenous genes may arise (Wade et al., 2005; Stringer et al., 2014). Although multiple two-component systems are usually well insulated from another (Skerker et al., 2008), interactions with endogenous cellular constituents cannot be ruled out *a priori* for these setups either.

### Optogenetic control upstream of transcription

Several strategies for the optogenetic regulation of bacterial expression act upstream of transcription initiation and control by light the availability of the DNA template to be transcribed (mechanism ❶ in Figure 3) or the activity of the RNA polymerase (mechanism ❷). Although light-regulated versions of the site-specific recombinase Cre were established in mammalian cells early on (Kennedy et al., 2010; Kawano et al., 2016; Taslimi et al., 2016; Meador et al., 2019; Morikawa et al., 2020), a corresponding system for bacteria arrived only more recently (Sheets et al., 2020). In all cases, the Cre recombinase is split into N- and C-terminal halves that by themselves have little mutual affinity and accordingly low catalytic activity. *Via* conjugation to photoreceptor pairs that associate under light, the fragments can be assembled, and recombinase action is restored. Whereas several photoactivable Cre recombinases for eukaryotic use rely on plant cryptochrome 2 (Cry2) and its interacting CIB protein (Kennedy et al., 2010), the prokaryotic setup harnesses the light-induced dimerization of *Nc*VVD or its Magnets derivatives (Sheets et al., 2020). The light-dependent activity of this system, called OptoCreVvd, was assessed with a reporter cassette comprising a transcriptional terminator flanked by *loxP* sites and followed by a gene encoding a red-fluorescent protein. Cre action promoted removal of the terminator sequence and hence led to an upregulation of reporter fluorescence by around 12-fold under blue light compared to darkness. Interestingly, the dynamic range of light regulation was higher when using the homodimerizing *Nc*VVD module rather than the heterodimerizing Magnets. Owing to the modularity of the setup, the design principle readily extended to the Flp recombinase which operates at the target FRT sites that are orthogonal to *loxP*. It is worth noting, that although light-induced reconstitution of the split recombinase fragments is fully reversible, the resultant recombination events are effectively irreversible under the chosen experimental conditions, which contrasts with essentially all other control points for the optogenetic regulation of bacterial expression. Depending on the application scenario, the effective irreversibility of the light-induced response can be advantageous. At the same time, irreversible systems generally mandate minimal basal (dark) activity, lest activation occurs prematurely. Even if low, basal activity might lead to gradual triggering of the optogenetic circuit to extents that will vary with the time that circuit is present in the bacteria.

Optogenetic control of bacterial expression was also accomplished at the level of RNA polymerase activity (mechanism ❷ in Figure 3) by two groups concurrently (Baumschlager et al., 2017; Han et al., 2017; Figure 4A). In both approaches, the activity of the viral T7 RNA polymerase (T7RNAP) was subjected to light control by fragmentation into two segments and linkage to the photoassociating Magnets or *Nc*VVD. Doing so allowed the upregulation of target-gene expression under blue light by up to several hundred-fold (Baumschlager et al., 2017), depending on the split site within the T7RNAP. Intriguingly, gene expression could also be upregulated by light to substantial degree if only one of the two T7RNAP fragments was ligated with either *Nc*VVD or one of the two Magnets (Han et al., 2017). Similarly, light-induced upregulation of gene expression resulted when said Magnet component was inserted into the T7RNAP between its N- and C-terminal halves. While maintaining stringent light responses, this design is realized as a single polypeptide component which should render its performance less dependent on its overall cellular concentration, see above. Taken together, the findings indicate that the *Nc*VVD LOV module and the derivative Magnets are capable of mediating different allosteric responses beyond mere dimerization. A later study also harnessed split T7RNAP to optogenetically regulate the expression of genes underlying lycopene biosynthesis in *E. coli* (Raghavan et al., 2020). In marked contrast to the earlier studies, T7RNAP was activated by red-light-induced intein splicing, akin to a previous implementation in yeast (Tyszkiewicz and Muir, 2008). To render protein splicing dependent on red light, a bipartite split intein was linked to the PCM of *A. thaliana* PhyB and its phytochrome-interacting factor (PIF) 3, respectively (Raghavan et al., 2020). Red light thus promoted assembly of the two split-intein components and allowed protein splicing to ensue. Using this strategy, unmodified and hence fully active T7RNAP could be obtained upon light-triggered intein processing. Light-induced activation in this manner is largely irreversible, excepting eventual T7RNAP turnover. Upon T7RNAP activation under red light, the lycopene production rose 5-fold. Beyond that, a key advance of the study is the functional expression of a plant phytochrome and its interacting factor in *E. coli*. Not only will this development pave the way towards further applications in bacteria, but also it stands to benefit the mechanistic study and possible modification of the PhyB:PIF interaction.

T7RNAP is an attractive target for optogenetic intervention as it recognizes promoters that are orthogonal to those served by the endogenous RNA polymerase (RNAP). By contrast, it is much more challenging to optogenetically control the bacterial RNAP to thus enable the light-dependent expression of a single or a few genes only. Although not realized to date, one principal avenue towards optogenetically regulating the bacterial RNAP could be the construction of light-regulated orthogonal sigma factors that recognize promoters not used by the endogenous sigma factors.

#### Optogenetic control of transcription

The vast majority of approaches for the optogenetic control of bacterial expression act at the level of transcription initiation (mechanisms ❸ and ❹ in Figure 3). Before treating them in detail, it is worth recapitulating basic aspects of the underlying processes (Müller-Hill, 1996). Transcription is initiated by promoter binding of the σ factor in complex with the RNAP, which in turn consists of two α and the β, β’, and ω subunits. Bacterial promoters are recognized by specific sequence motifs upstream of the first transcribed nucleotide, which is designated as the +1 position. Under normal conditions, the σ^70^ factor mediates the transcription of most genes in *E. coli* and other bacteria. The σ^70^ factor binds and thereby recognizes two conserved motifs centered around the −10 and −35 positions, with the former also known as the Pribnow box. Other σ factors differ in the sequence and precise location of their cognate operator motifs, thus enabling them to serve distinct sets of promoters. Once assembled at its promoter, the RNAP first dwells in its initiation mode and mediates repeated abortive transcription events. Only upon transitioning to its elongation mode, the RNAP clears the promoter and polymerizes mRNA in highly processive manner. The inherent strength of a given σ^70^-dependent promoter is largely governed by the sequences of the −10 and −35 boxes, with transcription usually the higher the closer these sequences are to the consensus motifs. However, even weak promoters commonly exhibit basal, if low, transcription levels in the absence of other factors (see Figure 2A). Transcription factors act by binding to specific operator sites near or within the promoters and thereby facilitate or hinder transcription initiation and elongation (Figures 3, 4B). Transcriptional activators often assemble on DNA stretches upstream of the −35 box and aid recruitment of the RNAP *via* productive interactions with the C-terminal domains (CTD) of the polymerase α subunits. Prominent examples include the catabolite activator protein (CAP) (Müller-Hill, 1996), also referred to as the catabolite repressor protein, and the l-arabinose-inducible AraC (Stringer et al., 2014). By contrast, bacterial repressors operate by interfering with binding of the σ factor and the RNAP, or with RNAP translocation and its processive mRNA synthesis. Compared to activators, repressors therefore exhibit more diverse locations of their operator sites, which are most frequently situated within or downstream of the promoter region. As a case in point, the well-known LacI repressor controls transcription of the *lac* operon *via* two operator sites upstream and downstream of the promoter in addition to the dominant operator site that interleaves with the promoter (Müller-Hill, 1996). Taken together, the effect of transcription factors on bacterial transcription is to some extent governed by where in relation to the transcription start site they bind. By the same token, transcriptional activators can be leveraged as repressors by judiciously moving their operator sites, as for instance shown for EL222 (Jayaraman et al., 2016; Ding et al., 2020) and CcaR (Ariyanti et al., 2021).

##### Two-component systems

As noted above, two-component systems are currently most widely used for the optogenetic regulation of bacterial expression (Figure 4C). Phosphorylation by the light-sensitive SHK generally activates the RR protein, frequently prompting its dimerization, and enables its binding to target operator sites. These sites are commonly located upstream of the −35 box and therefore allow productive interactions between the RR and the α-CTD of the RNAP. The first light-sensitive TCS suitable for optogenetics in bacteria was devised on the basis of the cyanobacterial Cph1 (Levskaya et al., 2005). Similar to pioneering work on light-inert, chimeric SHKs (Utsumi et al., 1989), the Cph1 PCM was covalently coupled to the effector unit of the *E. coli* EnvZ SHK which is engaged in osmosensing. In concert with the cognate RR OmpR, the resultant chimeric SHK Cph8 drove expression of a LacZ reporter in darkness, with an around 10-fold lower output under red light. As a cyanobacterial phytochrome, Cph8 required the provision of the PCB chromophore to elicit light responses, achieved *via* coexpression of the *ho* and *pcyA* genes (Tabor et al., 2011). Alternatively, the bilin chromophore might be exogenously added as routinely done for applications of plant phytochromes in yeast and mammalian cell culture (Shimizu-Sato et al., 2002; Levskaya et al., 2009; Müller et al., 2013). By optimizing the Cph8, HO, and PcyA expression levels and the target promoter sequence, the dynamic range of the Cph8:OmpR TCS was later improved to around 70-fold (Schmidl et al., 2014). The introduction of an inverter-gene cassette, based on the λ phage cI repressor and its target pR promoter (Elowitz and Leibler, 2000), reprogrammed the light response, resulting in higher expression under red light than in darkness (Tabor et al., 2011).

Next, the recombination of the LOV photosensor module of *B. subtilis* YtvA and the effector module of *Bradyrhizobium japonicum* FixL yielded the widely used, blue-light-responsive SHK YF1 (Möglich et al., 2009). In darkness, YF1 readily phosphorylates its cognate RR FixJ, also from *B. japonicum*, but under blue light the net kinase activity reduces by more than 1000-fold. The rather stringent response owes to the dual activity of YF1 as a net kinase in darkness and as a net phosphatase under blue light, respectively. The YF1:FixJ TCS achieved the downregulation of a LacZ reporter gene by around 70-fold under blue light in *E. coli* (Möglich et al., 2009). The flavin chromophore of YF1 is generally available in bacterial cells, which contrasts with the PCB chromophore utilized by Cph8 and CcaS (Tabor et al., 2011). Later on, the YF1:FixJ TCS was implemented on the pDusk plasmid that mediated the downregulation of a fluorescent reporter under blue light by around 10- to 15-fold (Ohlendorf et al., 2012). The light response of this TCS was inverted within the pDawn plasmid by the same λ cI-based gene cassette that successfully reprogrammed the Cph8:OmpR TCS (Tabor et al., 2011). Triggered by blue light, pDawn prompted an around 450-fold upregulation of expression. More recently, the pDawn system was expanded to the OptoLac setup for metabolic control in bacterial production processes (Lalwani et al., 2021a). In this system, the pDawn circuit was extended by an additional inverter cassette based on the *lac* repressor LacI and its operator *lacO*. As a result, the expression output was repressed by blue light, similar to but more efficient than the original pDusk. The dynamic range of light regulation in OptoLac was boosted to 60-fold by a negative feedback loop, in which LacI not only represses the target gene of interest but also the λ cI repressor (Lalwani et al., 2021a).

Analogous to the YF1 design (Möglich et al., 2009), the activity of the *Pseudomonas aeruginosa* GacS SHK was put under light control by exchanging its sensor domain for the LOV module from *B. subtilis* YtvA (Cheng et al., 2021). Use of the PATCHY method (Ohlendorf et al., 2016) facilitated the exploration of multiple SHK designs that differed in the length and sequence of the linker between the LOV photosensor and histidine-kinase effector modules. One variant, denoted YGS24, supported blue-light-activated phosphorylation of the GacA RR which, when phosphorylated, prompts the transcription of small regulatory RNAs in *P. aeruginosa* from specific promoters. Using a fluorescent reporter, the YGS24:GacA TCS mediated a 10-fold increase in gene expression from one of these promoters.

A widely used system for the optogenetic control of bacterial expression is based on the CBCR CcaS and its cognate RR CcaR which together control chromatic acclimation in *Synechocystis* sp. PCC6803 (Hirose et al., 2008; Hirose et al., 2010; Tabor et al., 2011). Transplanted into *E. coli*, the CcaRS TCS enabled the activation of target gene expression by green light which could be rapidly and completely reverted by ensuing illumination with red light. The initially modest regulatory response to green light of around 6-fold enhanced gene expression was subsequently boosted to more than 100-fold by adjusting the amounts of the TCS components and the promoter sequences, as also done for the Cph8:OmpR TCS (Tabor et al., 2011; Schmidl et al., 2014). An additional improvement of the light response arose from modification of the CcaS receptor itself which features two PAS domains between its CBCR photosensor and histidine-kinase effector modules. Removal of these two PAS domains, which are not known to respond to any signal, not only decreased the size of the resultant SHK, denoted mini-CcaS (Nakajima et al., 2016a), but also it further improved the regulatory response when embedded in a TCS together with CcaR. In the optimized setup (Ong and Tabor, 2018), target-gene expression increased by almost 600-fold under green light relative to darkness or red light. In addition to supporting high dynamic ranges, the CcaRS system offers the advantage of bimodal, photochromic control, see above. As a CBCR, CcaS requires the PCB chromophore which for bacterial expression is routinely provided by HO/PcyA coexpression. Intriguingly, length variations of the linker between the sensor and effector modules in mini-CcaS led to the generation of SHK variants that exhibited the opposite light response i.e., higher expression under red than under green light, albeit at somewhat reduced efficiency (Nakajima et al., 2016a). These observations resemble earlier findings for YF1, see above (Ohlendorf et al., 2016), and likely reflect SHK signal transduction *via* α-helical coiled coils (Möglich et al., 2009; Möglich, 2019).

As a group, CBCRs offer remarkably diverse color sensitivity, which can be in principle harnessed for bacterial optogenetics. As a case in point, the UirS CBCR SHK and its UirR RR, also from *Synechocystis* sp. PCC 6803 (Song et al., 2011), enabled the control of expression in *E. coli* by UV and green light (Ramakrishnan and Tabor, 2016). Irradiation with near-UV light around 380–400 nm engendered up to 6-fold enhanced target-gene expression which could be counteracted by green light. Although the dynamic range of regulation is comparatively low, it is important to note that the initial implementation of the CcaRS TCS showed light responses of similar magnitude (Tabor et al., 2011). Hence, there could be scope for much improving the extent of the UirRS light response along the lines previously successful for other systems (Schmidl et al., 2014).

We recently advanced derivatives of the pDusk and pDawn systems, dubbed pREDusk and pREDawn, that react to red and NIR, rather than blue light (Multamäki et al., 2022). To this end, the LOV module within YF1 was substituted for the PCM of the *Dr*BphP, thus yielding the new SHK *Dr*F1. Interestingly, target gene expression within the pREDusk system was decreased by around 200-fold under red light, thus much surpassing the blue-light response of the original pDusk. By contrast, pREDawn mediated an around 70-fold increase of gene expression under red light, which is somewhat less efficient than the pDawn performance (Ohlendorf et al., 2012). BphPs like *Dr*F1 require the supply of biliverdin as a chromophore which in pREDusk and pREDawn is ensured *via* coexpression of the *D. radiodurans* HO from within the same operon as the TCS.

##### Transcriptional repressors

A setup based on the bathyphytochrome BphP1 and the transcriptional repressor PpsR2, both from *Rhodopseudomonas palustris* CGA009, also employs two polypeptide components but is distinct from TCSs (Ong et al., 2018). When converted to its Pr state by far-red light, *Rp*BphP1 heterodimerizes with *Rp*PpsR2 and thereby impairs repression. The optimization of promoter sequences and expression levels led to an optogenetic system that achieved up to 2.5-fold upregulation of a fluorescent reporter under NIR light compared to red light or darkness. Despite a comparatively low dynamic range of regulation, the *Rp*BphP1:*Rp*PpsR2 system has the advantage of being activated by NIR light.

Compared to the previous systems, a series of setups achieve optogenetic control of bacterial expression by directly, rather than indirectly as in TCSs, controlling the activity of transcriptional activators and repressors. As noted above, these setups generally offer a simpler architecture and smaller genetic footprint than TCSs. Although most pertinent setups achieve optogenetic regulation *via* light-dependent dimerization and dissociation reactions, the pioneering LOV-TAP system does not but relies on other modes of allostery (Strickland et al., 2008). This system harnesses the widely used *As*LOV2 module which undergoes reversible unfolding of its N-terminal A’α and C-terminal Jα helices under blue light (Harper et al., 2003; Zayner et al., 2012; Dietler et al., 2022). Within LOV-TAP, the *As*LOV2 domain is fused to the *E. coli* tryptophan repressor (TrpR) such that the Jα helix overlaps in sequence with an N-terminal helix of TrpR. A scenario of mutually exclusive folding/function results, where either *As*LOV2 or TrpR, but not both entities, can claim the shared helical segment. In darkness, the helix is predominantly folded onto the *As*LOV2 core domain and hence not available to TrpR. Under blue light, the affinity of *As*LOV2 for its Jα helix drops, and the TrpR thus claims the shared helix and thereby becomes competent to bind DNA. The initially low blue-light-induced gain in DNA affinity of 6-fold was later improved to around 65-fold by modulating the interface between the *As*LOV2 core and the Jα helix (Strickland et al., 2010). Despite these advances, LOV-TAP has seen little use in bacterial optogenetics (Abbondanza et al., 2017), potentially because its overall DNA affinity is much reduced compared to the wild-type TrpR (Strickland et al., 2008; Strickland et al., 2010). That notwithstanding, LOV-TAP represents one of the pioneering examples that showcased how LOV domains can serve to regulate the activity of target proteins by light (Lee et al., 2008; Strickland et al., 2008; Möglich et al., 2009; Wu et al., 2009).

Building on the LightOn system for blue-light-activated mammalian gene expression (Wang et al., 2012), the LightOff setup mediates blue-light-repressed bacterial expression (Chen et al., 2016; Figure 3B). This setup employs the chimeric transcription factor LEVI, which comprises the homodimerizing *Nc*VVD module connected to the C-terminal DNA-binding domain (DBD) of the *E. coli* LexA repressor. LexA is an integral part of the UV-induced SOS stress response and regulates the expression of several target genes in *E. coli* (Wade et al., 2005). As the isolated LexA DBD is monomeric, it shows little affinity for its target operators. Linkage to *Nc*VVD and light-induced assembly restored the homodimeric state of the LexA DBD and DNA binding. In the LightOff system, LEVI achieved pronounced downregulation of a fluorescent reporter by around 10,000-fold under blue light. Remarkably, the reported regulatory effect thus significantly surpassed that for induction by IPTG (β-isopropyl-thiogalactoside) of a T7-*lacO* promoter, even when the T7 lysozyme was included *via* the pLysS plasmid (Ohlendorf et al., 2012; Chen et al., 2016). Apart from small-scale formats, LEVI also supported light-repressed gene expression at the fermenter scale. LEVI was further combined with a λ cI-based inversion cassette to furnish the LEVIon system which achieved a 500-fold upregulation of expression under blue light.

An advantageous property of the dimerization-based optogenetic strategies is their inherent modularity which facilitates the construction of derivative systems with novel properties. This was duly exploited for the development of the eLightOn setup which uses LexRO, a covalent fusion between the LexA DBD and the *Rs*LOV module (Li et al., 2020). In darkness, *Rs*LOV mediated homodimerization of LexRO and repression at target promoters; under blue light, LexRO dissociated into monomers, and the expression of a fluorescent reporter increased by up to around 500-fold. Notably, the LexRO setup utilizes the modified LexA_408_ variant with altered DNA specificity and reduced affinity for the endogenous bacterial LexA-dependent promoters (Thliveris et al., 1991). Use of this variant is thus expected to reduce off-target activity and limit the impact on endogenous pathways. The eLightOn setup enabled the regulation of bacterial motility and cell morphology as a function of blue light. Moreover, the combination of LexRO with the chemically inducible AraC yielded genetic circuits which acted as Boolean logic gates and achieved different outputs depending on the input signals blue light and l-arabinose. Notably, LexRO exhibited robust performance at 37°C which contrasts with a temperature lability of the wild-type *Rs*LOV module reported in other studies (Richter et al., 2016; Dietler et al., 2021).

Again using the LexA_408_ DBD, a red-light-responsive bacterial gene expression system was established (Kaberniuk et al., 2021). This system harnesses the light-induced oligomerization of a modified PCM from the *Idiomarina* sp. A28L BphP (*Is*PCM) that forms a homodimer in its Pr state but a homotetramer in its Pfr state. Linked to the LexA_408_ DBD, the *Is*PCM afforded the downregulation of a fluorescent reporter by 115-fold under red light, indicating that the chimeric transcription factor is more active in its tetrameric than its dimeric state.

The homodimeric Tet repressor (TetR) supports many applications in both prokaryotic and eukaryotic hosts, prominently so within the Tet-ON and Tet-OFF systems (Gossen et al., 1995). Although an early study subjected TetR-based mammalian expression circuits to optogenetic control, it did so by regulating in light-dependent manner the activity of a eukaryotic *trans*-activation domain appended to TetR (Müller et al., 2015). Consequently, the system did not translate to the prokaryotic setting. We recently developed a suite of light-regulated TetR variants based on light-induced homodimer association or dissociation (Dietler et al., 2021). Serial C-terminal truncation impaired TetR dimerization and incurred a loss of repression at target operators. The homodimeric state and repression capability were rescued by C-terminal fusion of different LOV modules. The dissociating *Rs*LOV underpins the pLITR system which prompted upregulation of a fluorescent reporter under blue light. While the regulatory efficiency at 29°C amounted to around 40-fold, it plummeted to around 2-fold at 37°C. The poor performance at the higher temperature could be tied to an overall low homodimer affinity in dark-adapted *Rs*LOV, see allosteric mechanisms above, and an intrinsic temperature lability. The performance at 37°C could be improved to up to ∼14-fold dynamic range in two *Rs*LOV variants harboring the D109G mutation or a redesigned dimer interface. As these modifications concern the *Rs*LOV module itself, the variants could also apply to other setups based on the same LOV module. As a case in point, these variants may benefit the LexRO system (Li et al., 2020), which at least in one instance failed to elicit light-induced expression changes (Wang et al., 2022), potentially due to the temperature lability of *Rs*LOV. Owing to the modular design, the *Rs*LOV module was easily exchanged for the associating *Nc*VVD and *Pt*LOV modules (Dietler et al., 2021). In the corresponding pLATR setups, TetR repression was enhanced by blue light, and up to 75-fold reduction of gene expression resulted. In another study, the repression by TetR was subjected to light control by splitting the repressor into two parts (Komera et al., 2022). Linkage of the resultant fragments to *Nc*VVD provided the TRU system which mediated the light-induced reconstitution of the repressor and a 13-fold downregulation of a fluorescent reporter. Combination with the LacI repressor generated the inverted TAU circuit in which target gene expression increased by up to 5-fold under blue light.

The above light-regulated transcriptional repressors are complemented by the CarH receptor from *Myxococcus xanthus* which in darkness binds as a homotetramer to target operators and thereby blocks expression (Ortiz-Guerrero et al., 2011). Green light drives the irreversible dissociation into CarH monomers which detach from DNA and relieve repression. Using LacZ as a reporter, green light thus elevated gene expression by around 10-fold. As pointed out above, the application of CarH outside its original host is complicated by the requirement for the cobalamin chromophore.

Beyond conventional repressors, the RNA-guided DNA endonuclease Cas9 can also mediate transcriptional repression once its catalytic activity has been disrupted by mutagenesis. In the so-called CRISPR interference (CRISPRi) strategy (Gilbert et al., 2013), the cleavage-deficient dCas9 serves as a programmable repressor that can be adapted to near-arbitrary DNA targets *via* single-guide RNAs (gRNA) of matching sequence. This key property was exploited in several studies that regulate the expression of dCas9 in light-dependent manner, rather than its activity (Wu et al., 2014; Wu et al., 2021; Zhang and Poh, 2018). Although several directly light-regulated (d)Cas9 variants were developed for mammalian use, they often achieve optogenetic regulation *via* light-dependent recruitment of transcriptional effector modules but leave sequence-specific DNA binding, central to CRISPRi, unaffected by light. That said, at least certain dCas9 variants that are regulated by light-dependent dimerization reactions should also apply to optogenetics in bacteria (Nihongaki et al., 2015; Richter et al., 2016; Zhou et al., 2018). Despite the potential of these approaches, they have to date seen little use in bacterial optogenetics and will hence not be treated in detail here.

##### Transcriptional activators

In addition to repressors, transcriptional activators were also leveraged for optogenetic expression control in bacteria. The LOV helix-turn-helix (HTH) receptor EL222 from *Erythrobacter litoralis* (Nash et al., 2011; Motta-Mena et al., 2014) that homodimerizes and binds to DNA when activated by blue light underlies several systems (Jayaraman et al., 2016; Ding et al., 2020; Camsund et al., 2021; Figure 4B). By placing the EL222 target operator upstream of the -35 promoter region, expression of a fluorescent reporter was ramped up by maximally 5-fold under blue light within the pBLind setup (Jayaraman et al., 2016). Within the pBLrep setup, said operator was placed between the -10 and -35 regions, and hence blue-light-induced EL222 binding caused a 3-fold reduction of gene expression. The availability of two compact EL222-based setups that elicit opposite outputs in response to light paves the way towards novel applications, as showcased for the light-dependent regulation of communication between bacterial cells (Jayaraman et al., 2016). A later study used a highly similar strategy to obtain the BLAT and BLRT systems for the light-induced 24-fold increase and 53-fold reduction, respectively, of bacterial gene expression (Ding et al., 2020). The better performance of BLAT and BLRT over pBLind and pBLrep owed to optimization of the EL222 expression levels and the sequence of its target operator site.

One of the most common systems for chemically inducing bacterial expression employs the l-arabinose (L-Ara)-responsive AraC transcriptional activator and its target P_BAD_ promoter. Removal of the N-terminal dimerization and L-Ara-binding domain of AraC rendered the C-terminal DBD monomeric and largely incapable of activating expression (Romano et al., 2021; Figure 4B). Similar to the LightOff setup (Li et al., 2020), the BLADE system restored the homodimeric state of the AraC DBD and transcriptional activation by N-terminal appendage of the *Nc*VVD module (Romano et al., 2021). Effectively, the chimeric AraC-*Nc*VVD transcription factor thus recapitulated the architecture and activation mechanism of the naturally occurring EL222 which also relies on light-activated dimerization (Nash et al., 2011). When exposed to blue light, BLADE triggered the upregulation of fluorescent-reporter expression by up to 15-fold. Commendably, the authors assessed in detail how the light-dependent response of BLADE scales with the expression strength of AraC-*Nc*VVD, that in turn governs its intracellular concentration. As fundamentally expected for setups that activate *via* dimerization, see Figure 2B and (Ziegler and Möglich, 2015), the performance of BLADE strongly depended on the AraC-*Nc*VVD levels (Romano et al., 2021). Whereas at intermediate expression levels, a substantial upregulation of expression could be induced by light, at lower or higher levels, the light response was partially or completely degraded. To at least certain extent, similar effects also apply to all other oligomerization-based optogenetic tools, although this aspect has seldom been investigated. With an optimal AraC-*Nc*VVD expression set by suitable constitutive or inducible promoters, robust light responses could be evoked by BLADE while maintaining low leakiness. Given the modular architecture of BLADE, the *Nc*VVD module could be functionally replaced by *Vf*LOV, albeit at lower efficiency. While *Nc*VVD performed better when connected to the N terminus of the AraC DBD, rather than the C terminus, the opposite proved true for *Vf*LOV. These findings arguably reflect the signal transduction mechanisms of these LOV modules which hinge on their N- and C-terminal segments, respectively.

##### Second-messenger signaling

Besides the above setups relying on TCSs and oligomerization reactions, a clade of systems achieve light-dependent bacterial expression *via* second-messenger signaling. Following their initial discovery in protists (Iseki et al., 2002), photoactivated adenylyl cyclases, that produce 3′,5′-cyclic adenosine monophosphate when exposed to light, were identified in different organisms (Ryu et al., 2010; Stierl et al., 2011; Raffelberg et al., 2013; Avelar et al., 2014; Ohki et al., 2017; Blain-Hartung et al., 2018) or were obtained by protein engineering (Ryu et al., 2014; Fushimi et al., 2017; Etzl et al., 2018; Stüven et al., 2019; Figure 4D). As exemplified for bPAC from *Beggiatoa* sp. (Ryu et al., 2010; Stierl et al., 2011), certain PACs exhibit stringently light-regulated cyclase activity with dynamic ranges of several hundred-fold. PACs can be harnessed for driving bacterial expression by combining them with the endogenous catabolite activator protein which acts as a transcriptional activator when in complex with cAMP. Although strong gene-expression responses can be thus evoked, the application scope of PACs appears limited given that cAMP serves as a general second messenger in many bacteria that triggers pleiotropic endogenous responses. A potential solution to this challenge may be provided by light-activated cyclases that produce 3′,5′-cyclic guanosine monophosphate (cGMP) (Etzl et al., 2018) and cGMP-responsive CAP homologs (Roychowdhury et al., 2015). That is because, in contrast to mammalian organisms, most bacteria do not employ cGMP for signal transduction.

Apart from cyclic mononucleotides, bacteria also use cyclic dinucleotides for signaling, most prominently 3′,5′-cyclic diguanylate. As exemplified by BphG1 from *Rhodobacter sphaeroides*, many bacteriophytochromes naturally regulate the activity of GGDEF effectors that synthesize c-di-GMP (Ryu and Gomelsky, 2014). The exchange of the original GGDEF module of BphG1 for a homolog generated BphS with improved light regulation of c-di-GMP synthesis. When coexpressing BphS with the c-di-GMP-dependent transcriptional activator MrkH from *Klebsiella pneumoniae*, the expression of a LacZ reporter could be enhanced by red light. The inclusion of a constitutively active EAL enzyme, which hydrolyzes c-di-GMP, reduced leakiness and thereby improved the dynamic range of the system to around 40-fold. Later on, the circuit was expanded by the blue-light-activated BLUF-EAL phosphodiesterase BlrP1 from *K. pneumoniae* (Huang et al., 2018), thereby enabling bimodal control of c-di-GMP synthesis and hydrolysis by red and blue light, respectively. Optogenetic circuits relying on light-dependent c-di-GMP production may further benefit from more recently engineered BphP-GGDEF variants that are regulated in their activity by up to 800-fold by red light (Gourinchas et al., 2019). As discussed for cAMP, c-di-GMP also triggers a raft of endogenous pathways, not least prompting the formation of biofilms in many bacterial species. Unless biofilm formation and other c-di-GMP-dependent processes are specifically demanded (Huang et al., 2018), the application of BphS to regulating bacterial gene expression may be restricted.

### Optoribogenetic control at the mRNA level

Whereas the above approaches regulate bacterial gene expression at the DNA level, several optoribogenetic setups operate at the mRNA level (mechanism ❺ in Figure 3). The first of these systems employs the LOV photoreceptor PAL from *Nakamurella multipartita* (Weber et al., 2019) which comprises a sequence-specific RNA-binding ANTAR effector (Shu and Zhulin, 2002). In darkness, the ANTAR moiety of PAL is in tight complex with a LOV module and thus autoinhibited. Blue light relieves the intramolecular inhibition and thereby increases the affinity of PAL for specific RNA hairpins, denoted aptamers in the following, by 100-fold or more to between 5 and 20 nM, depending on RNA sequence (Weber et al., 2019; Ranzani et al., 2022). The sequence-specific, light-activated RNA binding of PAL was leveraged for the regulation of bacterial expression by interleaving the aptamer with the Shine-Dalgarno (SD) sequence of a target gene (Figure 4E). Once activated by light, PAL could then bind to this region of the mRNA and thereby interfere with translation, which led to an up to 10-fold reduction of fluorescent reporter expression at 29°C (Weber et al., 2019). To facilitate the adoption of PAL-based optoribogenetic circuits, we recently developed the pCrepusculo system which is realized on a single plasmid and implements an improved aptamer sequence with higher affinity for PAL (Ranzani et al., 2022). pCrepusculo enabled the 8-fold downregulation of expression at 37°C. As for several of the transcription-based optogenetic approaches (Tabor et al., 2011; Ohlendorf et al., 2012), introduction of a λ cI repressor cassette inverted the response to light, and the resulting pAurora system prompted 67-fold increased gene expression under blue light (Ranzani et al., 2022). We recently also harnessed the light-induced PAL:aptamer interaction to repress by blue light the autocatalytic cleavage of a modified hammerhead ribozyme (HHR) (Pietruschka et al., 2022). By embedding the HHR in the 5′-untranslated region (UTR) of an mRNA, its SD sequence was masked, and expression thus attenuated. In darkness, the HHR can cleave itself and thereby expose the SD region, allowing translation to ensue. Light-induced binding of PAL to the modified HHR interfered with ribozyme cleavage and therefore led to an around 3-fold repression of bacterial expression under blue light. Although this optoribogenetic strategy currently has comparatively low regulatory efficiency, it hints at the versatility of light-dependent protein:RNA interactions. While the natural targets of PAL in *N. multipartita* have not been identified yet, it appears likely that the light-dependent regulation involves a transcriptional anti-termination mechanism, where binding of the ANTAR protein to the nascent mRNA disrupts an intrinsic terminator sequence (Wilson et al., 1993). By that token, PAL can presumably support yet other modes of optoribogenetic regulation at the mRNA level.

The more recent LicV setup indeed employs transcriptional antitermination and thereby upregulates bacterial gene expression by around 17-fold under blue light (Liu et al., 2022). LicV is based on the co-antitermination (CAT) domain of *B. subtilis* LicT which is monomeric in isolation and hence little active. Connection to a C-terminal *Nc*VVD module enabled light-induced homodimerization, followed by CAT binding to the target RAT motif in the nascent mRNA and antitermination. The affinity of LicV for its target RAT motif amounted to 90 nM in blue light and around 3.8 µM in darkness, corresponding to an around 42-fold-difference. Compared to PAL, the binding of LicV to its RNA is thus weaker and less strongly regulated by blue light (Weber et al., 2019; Ranzani et al., 2022).

The optoribogenetic regulation of bacterial expression has several traits that may prove advantageous to application. First, as the light-dependent regulation is exerted at the mRNA level, the corresponding setups lend themselves to combinations with circuits, optogenetic or otherwise, that act at the DNA level (Ranzani et al., 2022). Doing so may give rise to integrated circuits with finer-grained and more pronounced light responses, which potentially benefit optogenetic applications, for example in bioproduction processes, see below. Second, as not least illustrated by the highly versatile bacterial riboswitches (Breaker, 2018), signal-dependent RNA interactions can be leveraged in multiple ways for expression control. For example, the stability of certain mRNAs in *E. coli* and hence the expression of proteins encoded by them is regulated by binding of proteins to cognate RNA motifs within the 3′-UTR (Tang and Guest, 1999). Although not realized yet, integrating the RNA aptamers recognized by PAL and LicV into these motifs may render mRNA stability and gene expression light-dependent.

#### Posttranslational optogenetic control

The steady-state intracellular activity and concentration of target proteins may also be optogenetically regulated after translation is completed. While versatile optogenetic strategies now allow the direct allosteric light-dependent control of various proteins (Dagliyan et al., 2016; Losi et al., 2018), they are often particular to the protein in question and require its covalent modification with light-sensitive photoreceptor modules. In this treatise, we will hence not consider these specific strategies, but rather focus on generic approaches that apply to various targets with minimal adaptation necessary. As one example, several optogenetic strategies modulate protein activity *via* light-dependent sequestration into clusters or complexes. For instance, two approaches based on plant cryptochromes and cyanobacterial BLUF receptors, respectively, enable the formation or dissolution of protein clusters and RNA-dependent liquid-liquid phase separation (LLPS) upon illumination (Shin et al., 2017; Dine et al., 2018) (mechanism ❻ in Figure 3). Ligation with photoreceptor modules engaged in clustering allows the light-dependent removal of target proteins from the regular cytosolic pool and their sequestration into the separated liquid phases. Inside these membrane-less organelles, the activity of the protein of interest may be lowered. Alternatively, enzymes may thus be colocalized, and metabolic flux increases, as demonstrated in yeast (Zhao et al., 2019). Several recently developed classes of light-activated nanobodies and monobodies may find similar application for the sequestration and colocalization of target proteins (Reis et al., 2018; Yu D. et al., 2019; Carrasco-López et al., 2020; Gil et al., 2020; He et al., 2021; Woloschuk et al., 2021). Moreover, light-activated binding to the protein of interest may directly reduce its activity.

Finally, the intracellular lifetime of target proteins can be optogenetically controlled (mechanism ❼ in Figure 3). Corresponding strategies are well established in eukaryotes (Renicke et al., 2013; Bonger et al., 2014) and involve the light-controlled unmasking of a degron signal which then prompts protein degradation *via* the ubiquitin-proteasome system. By contrast, optogenetic strategies for the deliberate, light-induced degradation of target proteins in bacteria are scarce. In *E. coli*, truncated proteins arising from incomplete translation are marked by a C-terminal peptide tag that is encoded by the *ssrA* transfer-messenger RNA. Proteins are thus designated for degradation by the ClpAPX protease. This principle has long been exploited to control the persistence of proteins inside bacteria (Andersen et al., 1998). The intracellular lifetime can be greatly modulated by appending to target proteins different variants of the *ssrA* peptide tag, with the three most C-terminal residues being particularly important. Interestingly, one of the most widely used setups for light-induced protein:protein interactions, the iLID system (Guntas et al., 2015), employs a modified *ssrA* peptide that lacks these three C-terminal residues. It has however not been reported if or to what extent iLID can be reconfigured for inducing protein degradation by light in bacteria. Against this backdrop, the PRU approach pursues a different strategy based on split TEV protease and the *Nc*VVD module (Komera et al., 2022). Blue light prompted reconstitution of the TEV protease and enabled the on-demand cleavage of target proteins. When the specific TEV target epitope was incorporated into a fluorescent reporter, blue light triggered a 12-fold reduction in fluorescence. The response to light could be inverted by appending to the C-terminus of target proteins a *ssrA* peptide tag *via* a linker containing a TEV target site. In this manner, the split TEV protease cleaved off the tag under blue light, the degradation *via* the ClpAPX system was reduced, and the reporter fluorescence increased 4-fold.

## Multiplexed control of bacterial expression

The cohort of available optogenetic tools, summarized above, also support multiplexed control of gene expression by several stimuli rather than a single light color. For an in-depth overview on multiplexed optogenetic circuits, we refer to (Dwijayanti et al., 2022). Multiplexing can potentially provide more stringent light-dependent regulation and better resolution of gene expression in time and space (Ziegler and Möglich, 2015). While not relevant for every single of the applications detailed below, certain use cases of light-regulated bacterial gene expression could benefit from these aspects. The arguably most straight-forward option for implementing multiplexed optogenetic control uses photochromic photoreceptors, particularly those of the phytochrome superfamily, that are inherently sensitive to two light colors. As noted above, in these photoreceptors not only the population of the signaling state is light-driven, but also its depletion is. The reversion to the resting state can thus be much accelerated compared to non-photochromic receptors which feature slow, thermal recovery reactions (Figures 2E,F). The bidirectional switching afforded by photochromic photoreceptors thus enables superior temporal precision and allows gene expression to be toggled on and off at will, even repeatedly. Beyond photochromic systems, certain photoreceptors combine multiple photosensor modules into a single polypeptide, arguably to sense and integrate several light stimuli. Apart from the well-known neochromes (Nozue et al., 1998) and *Rhodospirillum centenum* Ppr (Jiang et al., 1999), neither of which (yet) appears to immediately apply to the optogenetic expression control in bacteria, it is foremost the CBCR receptors that are relevant. These receptors often comprise arrays of precisely spaced CBCR modules that individually respond to distinct light colors (Rockwell et al., 2013; Fushimi and Narikawa, 2019). Likely, composite CBCR receptors register several light cues and compute a joint output signal. Receptors that comprise several sensor domains and integrate (light) stimuli can also be engineered, as exemplified for SHKs that respond to both light and oxygen levels (Möglich et al., 2010).

As an alternative to photochromic and multi-sensor receptors, certain photoreceptors and derived circuits are either inherently sensitive to other signals besides light or can be configured thus. With such setups, gene expression may be controlled more precisely than possible for simpler circuits that obey one signal only (Figures 2E,F). To this end, several studies combined optogenetic circuitry with chemically inducible transcription factors e.g., LacI or AraC, (Jayaraman et al., 2018b; Li et al., 2020), to construct Boolean logic gates that controlled gene expression jointly by light and chemicals. In a similar vein, the BLADE approach subjected the activity of the AraC DNA-binding and activation domain to optogenetic control (Romano et al., 2021). When combining BLADE with the wild-type AraC, target genes could be induced by either blue light or l-arabinose. Moreover, certain photoreceptors are sensitive to small ligands in addition to light. As the above-described pLITR and pLATR systems retain the ligand-binding portion of the parental TetR, they can be toggled not only by light but also by tetracycline analogs, which could be harnessed for fine-grained gene-expression control (Dietler et al., 2021). For instance, repression within the pLATR setup might first be activated by blue light, hence leading to reduced gene expression; subsequent addition of anhydrotetracyclin would prompt repressor dissociation and restoration of gene expression. Such a setup could be expanded even further as tetracycline is inherently light-sensitive which was exploited for the (non-optogenetic) light-dependent control of bacterial expression (Baumschlager et al., 2020). Moreover, certain pLITR variants based on wild-type *Rs*LOV are labile to temperature increases which could potentially be leveraged for multiplexed control of gene expression by light and temperature (Dietler et al., 2021). Although the integration of light signals with other chemical and physical cues can offer certain benefits, there are also limitations to these approaches. Given that systems like the above depart from purely light-dependent control, the desirable traits of optogenetics, such as reversibility, spatiotemporal precision, and non-invasiveness, may be degraded.

Multiplexed control can also rely on combinations of several optogenetic tools for bacterial expression. Depending on application, the tools might either be used in parallel in unmodified form, or they may be integrated into joint circuits. As an example of the former, EL222 can serve as either an activator or a repressor, and at different promoters, gene expression can be thus either up- or downregulated by blue light (Jayaraman et al., 2016; Ding et al., 2020). The latter approach appears most straight-forward for pairs of optogenetic implements that act at distinct levels of the gene-expression trajectory, for example at the DNA and mRNA levels, respectively (Ranzani et al., 2022). When multiple photoreceptors are used in concert within the same bacterium or within co-cultures of different bacteria, it is desirable to toggle them by light independently from another. Several options are available to this end, with the most obvious being the use of photoreceptor pairs that are sensitive to different light colors. For instance, one of the many blue-light-sensitive setups may be combined with the CcaRS system (Fernandez-Rodriguez et al., 2017). As is apparent from the absorbance spectra of CcaR in its Pg and Pr states (Figure 1B), to some extent this photoreceptor also absorbs blue light which may principally trigger its interconversion. However, even if inadvertent activation by blue light occurred, subsequent irradiation with red light could counteract this effect. Moreover, we recently demonstrated that the pREDusk and pREDawn platforms, that are based on a BphP, are readily switched by red light but relatively insensitive to comparable intensities of blue light (Multamäki et al., 2022).

By contrast, if two photoreceptors respond to the same light bands e.g., pairs of LOV receptors, they cannot be spectrally separated. However, even then the individual receptors may still be sequentially addressed if they sufficiently differ in their light sensitivity. At low light doses, only the more sensitive circuit would be triggered by light, before at higher light doses the second circuit kicks in as well. Given the hyperbolic dose-activation profiles that many photoreceptor circuits exhibit, see above, the full separation of two systems in this manner may however be difficult (Ziegler and Möglich, 2015; Hennemann et al., 2018; Figure 2A). Separation becomes easier for optogenetic circuits that respond to light cooperatively. The resultant sigmoidal activation profiles may be more readily separated in the intensity regime (Figure 2A). Alternatively, two optogenetic circuits may be sequentially toggled if they have sufficiently different recovery time courses after triggering by light (Hennemann et al., 2018; Figures 2C,D). In that scenario, the circuits can be sequentially addressed as they react differentially to trains of light pulses of suitable temporal spacing. The circuit with the faster recovery reaction dwells for less time in the signaling state than the other circuit before reverting to its resting state. Put another way, all other parameters being equal, the circuit with the slower recovery is activated to larger extent than the one with the faster reversion. Based on this principle, two variants of the pDawn circuit that differed in their dark-recovery kinetics could be toggled sequentially by blue light (Hennemann et al., 2018).

Not only multiplexed approaches, but also other optogenetic applications benefit from the online monitoring of the system under study, for instance *via* continuous measurements of reporter fluorescence or cell density. Such information can be used to infer the current state of the optogenetic circuit(s) and to suitably adapt the light intensity (or, color) to maintain or alter the system state as demanded by application. These approaches are particularly effective for photochromic receptors as their activity state can be bidirectionally changed by different light colors, as demonstrated for the widely used CcaRS TCS (Davidson et al., 2013; Milias-Argeitis et al., 2016; Chait et al., 2017; Steel et al., 2020). However, in principle other optogenetic circuits may also be controlled in feedback manner, as recently shown for pDusk, pDawn (Datta et al., 2022), and light-responsive T7RNAP (Gutiérrez Mena et al., 2022).

### Applications of optogenetic expression control in bacteria

**FIGURE 5:**
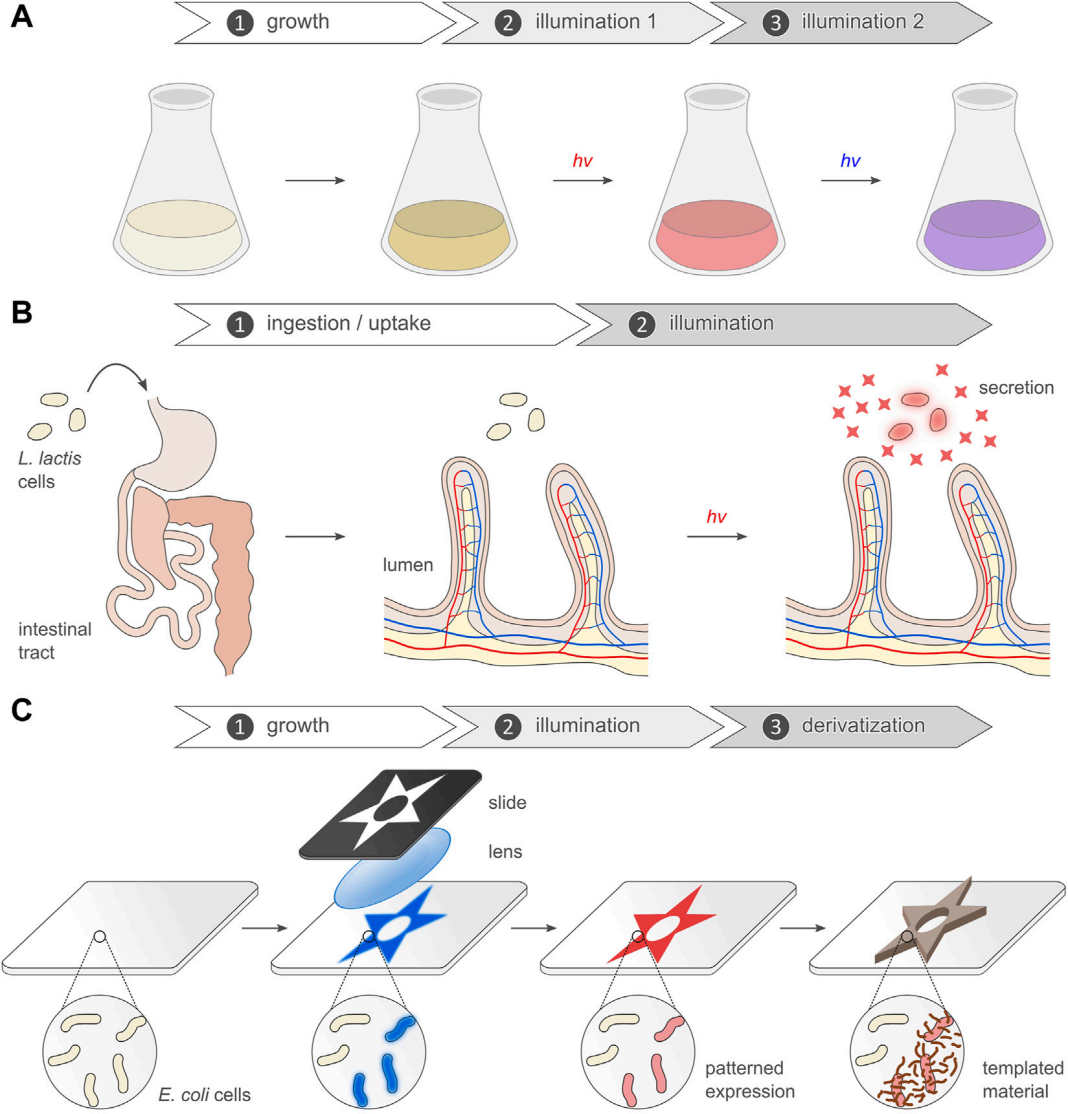
Use cases of optogenetic expression control in bacteria. **(A)** Light-dependent gene expression underpins the regulation and optimization of bioproduction processes. The dynamic control afforded by optogenetics for instance allows the separation in time of growth and production phases. In a two-stage fermentation process, biomass can be first accumulated before illumination starts and production ramps up (Montaño López et al., 2022). The fundamental approach extends to systems that respond to several light colors to for example turn on and off the expression of target genes on demand. Optogenetic actuation may be combined with online (optical) monitoring of the system state, thus allowing continuous feedback control of the system (Milias-Argeitis et al., 2016). **(B)** Optogenetics serves to control by light gene expression in bacteria residing inside the body of animals e.g., within the intestinal tract. This strategy for example enables the optogenetic stimulation of the bacterial production and secretion of choice hormones or chemicals that bestow health benefits on the animal host (Hartsough et al., 2020; Yang et al., 2020). **(C)** Owing to the spatial precision of optogenetics, light-regulated bacterial expression lends itself to the production of structured biomaterials. The pertinent studies commonly expose lawns of bacteria to patterned illumination which elicits the spatially confined expression of target genes. Beyond enabling the so-called “bacterial photography” (Levskaya et al., 2005), the concept can be adapted for material production. For instance, light can prompt the bacteria to form biofilms which can be mineralized with inorganic compounds to thus yield composite living materials (Wang et al., 2021).

The past years have seen a growing number of studies capitalize on the above optogenetic tools and regulate bacterial expression by light (Figure 5). Whereas the initial implementation and subsequent deployment of most tools were in *E. coli* laboratory strains, increasingly applications address other bacteria, too. These reports suggest that at least to some extent the pertinent optogenetic circuits generally apply and translate to other microorganisms. Beyond *E. coli*, the widely used CcaRS TCS (Tabor et al., 2011) enabled light-regulated gene expression in *Synechocystis* cyanobacteria (Abe et al., 2014; Miyake et al., 2014; Badary et al., 2015) and *P. aeruginosa* (Hueso-Gil et al., 2020). Likewise, the pDawn setup (Ohlendorf et al., 2012) underpinned applications in the probiotic *E. coli* Nissle 1917 strain (Magaraci et al., 2014; Alizadeh et al., 2020; Cui et al., 2021), *P. aeruginosa* (Pu et al., 2018), the marine bacterium *Vibrio natriegens* (Tschirhart et al., 2019; Wang et al., 2020), and *Shewanella oneidensis* (Zhao et al., 2022). Similarly, EL222 was used in *Sinorhizobium meliloti* (Pirhanov et al., 2021). Applications of none of these three optogenetic circuits are restricted to Gram-negative bacteria but extend to for instance bacilli e.g., *B. subtilis* (Castillo-Hair et al., 2019), and *Lactococcus lactis* (Pan et al., 2021; Pan et al., 2022; Zhang et al., 2021). While in most of these studies the optogenetic setups were used essentially unmodified, other reports required the optimization of plasmid backbones, promoters, ribosome-binding sites, and chromophore supply to elicit and boost light-dependent gene-expression responses (Castillo-Hair et al., 2019; Hueso-Gil et al., 2020).

We loosely assign the increasingly diverse studies that employ light-regulated bacterial expression to four application categories: photosensing; bioproduction (Figure 5A); theranostics (Figure 5B); photography and templated materials (Figure 5C). We note that the individual categories are not mutually exclusive, with certain studies falling into more than one. As discussed in the following, the individual application categories capitalize on the benefits of optogenetic regulation in different fashion and degree.

#### Photosensing

Applications within this heterogenous group harness light-regulated gene expression for controlling bacterial physiology or for biosensing. These applications therefore primarily exploit the temporal dimension of optogenetics, while the spatial resolution and reversibility are of subordinate significance. For instance, to showcase the utility of LexRO (Li et al., 2020) and BLADE (Romano et al., 2021), respectively, both setups were deployed to render the expression of FtsZ blue-light-dependent and to thus achieve control of bacterial cell division. In a similar vein, LexRO served to regulate CheZ expression and to thereby modulate bacterial chemotaxis (Li et al., 2020).

Beyond proof-of-principle demonstrations, the CcaRS TCS enabled the optogenetic control of asymmetric cell division in *E. coli* (Mushnikov et al., 2019). Under green light, the bacteria expressed a fusion protein encompassing a c-di-GMP-degrading phosphodiesterase and the scaffold protein PopZ from *Caulobacter crescentus*. Notably, PopZ spontaneously concentrates in one cell pole within the heterologous *E. coli*, and the linked phosphodiesterase hence depleted c-di-GMP levels predominantly at this pole. Upon cell division, two daughter cells with different c-di-GMP concentrations arose. The inequal second-messenger concentrations in turn ushered in distinct downstream responses in the cells. The optogenetic regulation of engineered asymmetric cell division will not only facilitate mechanistic research but may also be relevant for biotechnology. For instance, the light-induced formation of cell progeny specializing on different aspects of a joint bioproduction chain could benefit multi-stage bioproduction processes (Mushnikov et al., 2019).

Apart from its use in basic research, light-regulated gene expression also supported innovative applications for biosensing in bacteria. In the SCRIBE system for cellular memory (Farzadfard and Lu, 2014), suitably configured *E. coli* bacteria “remembered” the exposure to external stimuli. To this end, the bacteria expressed the β recombinase from the λ phage in combination with a so-called retron cassette. This retron relies on a bacterial reverse transcriptase to achieve the *in situ* production of single-stranded DNA (ssDNA) oligonucleotides. In concert with the β recombinase, these oligonucleotides prompt the introduction of mutations at the site(s) specified by the ssDNA. The pDawn circuit was used to control the expression of a retron cassette directed against a deliberately incapacitated kanamycin resistance marker. Light-induced retron action thus eventually restored the resistance marker, which in turn could be quantified at the population level by antibiotics selection. Taken together, SCRIBE thus enabled the detection of blue-light stimuli with persistent memory.

A similar concept was pursued in the CAMERA approach (Tang and Liu, 2018). Among other strategies advanced in this study, the cleavage-deficient dCas9 was connected to a base-editing enzyme (BE) that promoted the introduction of mutations at target sites specified by gRNAs. Again, pDawn was used for controlling the expression of the dCas9-BE. Across a bacterial population, blue-light exposure thus gradually translated into persistent changes of genomic DNA sequence which could be captured by sequencing. The response at the DNA sequence level scaled with the extent of blue-light application, and the CAMERA approach could thus be used to count illumination events. Intriguingly, the setup achieved quantifiable light-dependent responses even if only ten bacteria were sequence-analyzed.

#### Bioproduction and metabolic engineering

The common denominator of applications within this large group is the light-dependent regulation of bacterial expression for bioproduction purposes (Hoffman et al., 2022; Montaño López et al., 2022). In the simplest case, the desired product is a protein or peptide, and its expression can then be directly controlled by light (Figure 5A). However, the basic approach also lends itself to the precise modulation of metabolic production processes that generate desired low-molecular-weight compounds. Irrespective of the specific scenario, applications within this group usually harness the temporal control, noninvasiveness, and reversibility afforded by optogenetics, whereas the spatial definition is often little or not important.

The original implementation and quantitative characterization of the available optogenetic circuits generally entailed the light-dependent expression of reporter genes, particularly fluorescent proteins (see Table 1). By that token, all these optogenetic systems are principally suited to controlling the production of target proteins, if by different regulatory mechanisms and with different effectiveness. Beyond small-scale expression, the original reports on certain optogenetic circuits also demonstrated the feasibility of light-regulated heterologous gene expression at the preparative and fermenter scales (Ohlendorf et al., 2012; Chen et al., 2016; Lalwani et al., 2021a). Although these experiments again involved reporter genes to aid detection and quantification, the optogenetic expression control clearly extends to near-arbitrary target proteins. This notion is borne out in several studies that used the pDusk/pDawn (Wu et al., 2014; Chang et al., 2017) and LEVI systems (Yu S. et al., 2019) to regulate by light the production of enzymes in *E. coli*. Light-controlled gene expression was also extended to *in vitro* expression systems (Jayaraman et al., 2018a; Zhang P. et al., 2020). In one study, purified EL222 and a DNA template, based on the pBLind system and encoding a red-fluorescent protein, were added to cell lysate for *in vitro* protein expression. Blue light induced an up to 10-fold increased fluorescence readout. When EL222 was encoded on the same DNA template, rather than added as purified protein, the dynamic range degraded to about 3-fold. Another study implemented the YF1:FixJ TCS to downregulate target genes by blue light in a cell-free system (Zhang P. et al., 2020); an inverter cassette based on the λ phage cI repressor achieved upregulation of expression by blue light. By expressing YF1 and FixJ individually in separate lysates, their ratios could be varied and thus optimized. Doing so culminated in the blue-light-mediated downregulation and upregulation of fluorescent reporters by maximally 6-fold and 3.5-fold, respectively. As noted by the authors, these systems may not only be interesting for *in vitro* protein production *per se* but also for engineering artificial cell-free systems with signaling capability.

Beyond macromolecular protein targets, many studies using light-regulated bacterial expression aim at optimizing the bioproduction yields of small metabolites and compounds. As a general strategy, such compounds may be synthesized by diverting intermediates from the cellular metabolism towards enzymatic pathways leading to the desired substance, as demonstrated in pioneering work in baker’s yeast (Zhao et al., 2018). In the pertinent studies, the redirection of metabolic flux is mostly achieved by optogenetically regulating the expression of key enzymes catalyzing chemical conversions at metabolic branchpoints. Reaction pathways are thus opened up or shut down in light-dependent manner. In a comparatively simple implementation of this concept (Wang et al., 2020), the pDawn circuit drove the expression of a tyrosinase in the fast-growing marine bacterium *V. natriegens* under blue light. The tyrosinase catalyzed the oxidation of tyrosine to *ortho*-dihydroxyphenylalanine (Dopa) and dopaquinone which in turn polymerized to the photoprotective melanin pigment. The optogenetic circuit thus installed temporal control of melanin pigment formation which may benefit industrial processes, according to the authors.

Two studies leveraged the CcaRS TCS to redirect the metabolic flux of glycolysis intermediates in *E. coli* (Senoo et al., 2019; Tandar et al., 2019). In one study (Tandar et al., 2019), the expression of glucose-6-phosphate (G6P) isomerase (GPI) was placed under CcaRS optogenetic control in a *gpi* knockout strain. Green light activated GPI expression and mediated isomerization of G6P to fructose-6-phosphate, thus allowing its further metabolization to pyruvate. By contrast, red light lowered GPI expression, and G6P was thus metabolized to bigger extent *via* the pentose-phosphate pathway that generates reduction equivalents in the form of NADPH. As bioproduction processes often involve the reduction of precursors to less oxidized, desired reaction products, the optogenetically controlled switch between glycolysis and pentose-phosphate pathway may prove widely useful. A second report targeted a step further downstream in glycolysis, namely the reversible interconversion of dihydroxyacetone phosphate (DHAP) and glyceraldehyde-3-phosphate (GAP), as catalyzed by triose-phosphate isomerase (TIM) (Senoo et al., 2019). When put under CcaRS control in a *tim* knockout background, TIM expression was turned on by green light, and glycolysis proceeded. Exposure to red light reduced TIM expression and hence caused DHAP accumulation as its isomerization to GAP was hampered. Under these conditions, DHAP was converted to pyruvate *via* the methylglyoxal (MGO) pathway, thus incurring increased levels of the eponymous MGO. Given that elevated MGO levels are cytotoxic, the authors argued that this mode of metabolic rewiring may be used for restricting growth and cell division in bioproduction processes, also see below.

A recent report combined the CcaRS and YF1:FixJ TCSs to shuttle metabolic flux between the tricarboxylic acid cycle (TCC) and production of polyhydroxybutyrate, a biodegradable polymer (Wang et al., 2022). To this end, the CcaRS system controlled the expression of citrate synthase (*gltA*) which is responsible for importing acetyl-CoA into the TCC. The light response of the YF1:FixJ TCS was inverted using a gene cassette based on the PhlF repressor (Stanton et al., 2014) and controlled the *phbABC* gene cluster that mediates PHB biosynthesis starting from acetyl-CoA. As the two optogenetic circuits respond to different light colors, the expression of the target genes could be controlled individually and thereby synchronized in time. The maximal PHB yield resulted when cultures were first grown in green light (i.e., expression of *gltA* but not *phbABC*), followed by sequential illumination with blue light (to activate *phbABC* expression), and replacement of green by red light (to shut off *gltA* expression).

The OptoLAC circuit, described above, was conceived for optogenetically regulating bioproduction processes in *E. coli* (Lalwani et al., 2021a). On the one hand, OptoLAC served to control the expression of a three-gene pathway that mediates the conversion of acetyl-CoA to mevalonate, an isoprene precursor. Bacteria harboring this system were first cultivated under blue light, thus shutting off target-gene expression. At a certain optical density, the cultures were transferred to darkness which prompted the gradual induction of expression and mevalonate production. Upon optimizing the timing of optogenetic induction control, mevalonate titers could be achieved that exceeded those obtained for chemical induction by around a quarter. In another application, OptoLAC controlled the expression of five genes that jointly catalyze the conversion of pyruvate to isobutanol. As before, bacterial cultures were first grown under blue light before transfer to darkness. Doing so led to maximal isobutanol yields that again were a quarter higher than when using chemical induction. A recent study (Cheng et al., 2022) employed OptoLAC to modulate the expression of a modified variant of fatty-acid photodecarboxylase from *Chlorella variabilis* (*Cv*FAP) (Sorigué et al., 2017). Notably, *Cv*FAP and its variants serve as photoenzymes that catalyze the blue-light-driven decarboxylation of fatty acids, α-hydroxy carboxylic acids, and α-amino acids. Owing to its stereospecificity, the *Cv*FAP variant can specifically decarboxylate the D isomer of phosphinothricin (PPT), thus enabling the production of the L isomer starting from a racemic D/L mixture. Cultivation under intermittent blue-light illumination toggled *E. coli* bacteria harboring OptoLAC-controlled *Cv*FAP between expression of the photoenzyme (darkness) and photocatalysis (blue light). Using this strategy, L-PPT was obtained in a one-pot reaction with better yield and enantiomeric excess than when illumination was continuous.

At least certain of the above examples required knockout bacterial strains to redirect metabolic flux. As demonstrated for the production of muconic acid, a precursor for chemical synthesis, the combination of optogenetic expression control and CRISPRi can potentially obviate this requirement (Wu et al., 2021). The EL222 circuit controlled the expression of cleavage-deficient dCpf1 and an associated array of gRNAs, thus enabling the knockdown of target genes upon blue-light exposure. With this strategy, several metabolic pathways that consume phosphoenol pyruvate could be inhibited, and metabolic flux could be diverted from the biosynthesis of aromatic amino acids to that of muconic acid. In addition to enabling the expression control of genomically encoded genes, rather than plasmid-borne ones, the CRISPRi strategy offers the advantage of hitting several targets simultaneously *via* different gRNAs.

Bioproduction processes and yields can also be enhanced by optogenetically controlling bacterial proliferation (Ding et al., 2020). To this end, one report combined the EL222-based BLAT and BLRT setups with a BphS-dependent, red-light-responsive circuit, denoted NRAT, to manipulate the timing and duration of bacterial DNA replication and cell division. Whereas the light-gated expression of ribonucleotide reductase promoted DNA synthesis, expressing FtsA and FtsZ accelerated cell division. Optogenetically regulating these and several other genes allowed the proliferation speed of *E. coli* to be set. By shortening the time taken for cell division, the production yield of the food flavor acetoin was improved. Contrarily, the optogenetically induced lengthening of cell division improved the yields of the biodegradable polymer poly-(lactate-co-3-hydroxybutyrate).

Certain bioproduction processes may benefit from co-culturing different microorganisms that jointly synthesize the compound of interest by “division of labor” (Lalwani et al., 2021b). Although desirable, the stable maintenance of microbial consortia over time is demanding, as one microorganism may outgrow other ones in the system. To address this challenge, the OptoTA setup harnessed the pDusk and pDawn circuits to antagonistically express the toxin-antitoxin MazF:MazE pair. *E. coli* cells equipped with OptoTA were hampered in their growth in darkness owing to MazF expression. Blue light repressed MazF expression, instead induced expression of the MazE antitoxin, and in sum thus promoted bacterial proliferation. In this way, *E. coli* and *Saccharomyces cerevisiae* could be co-cultured at desired titers which enhanced the bioproduction yields of isobutyl acetate (used as a solvent and potential biofuel) and naringenin (e.g., used as an antibiotic) (Lalwani et al., 2021b). Other means of optogenetically controlling bacterial viability and proliferation may also apply to microbial co-cultures. First, the above-described rewiring of glycolysis increased the levels of the cytotoxic MGO which may reduce cell viability and proliferation (Tandar et al., 2019). Second, the pDusk setup was used to express the phage-21-derived lysin and to thereby hamper cell proliferation in darkness. Blue light shut off lysin expression and thus accelerated proliferation (Wang G. et al., 2018). Third, a recent study detailed the application of OptoCreVvd to control the expression of antibiotic resistance genes by blue light (Sheets and Dunlop, 2022). Following light-induced DNA recombination, *E. coli* bacteria withstood antibiotics treatment which could prospectively represent a means of controlling cell titers in bioproduction processes.

#### Theranostics and towards biomedical applications

Applications within this category have in common that they harness light-regulated bacterial expression in diagnostic and therapeutic settings. The studies in question rely on the non-invasiveness and temporal precision afforded by optogenetics, whereas the reversibility and spatial precision of expression play minor roles to date. The non-invasiveness is especially important for use cases that envision or even realize the deployment of light-responsive bacteria inside the body of animals e.g., within their digestive tract (Figure 5B). As discussed above, the *in situ* light delivery for triggering the underlying optogenetic circuits may become limiting in such applications.

Various bacteria produce peptide- or protein-based cytotoxins capable of killing mammalian cells. By subjecting toxin expression to optogenetic control, bacteria may hence serve as light-responsive agents for the targeted destruction of mammalian cells, which could prospectively apply to cancer therapy. One early approach used the pDawn circuit in *E. coli* Nissle 1917 to express cytolysin A, a cytotoxin made by different *Enterobacteria* (Magaraci et al., 2014). When exposed to blue light, the bacteria responded by cytolysin production which caused the lysis of red blood cells in blood agar. By contrast, no cytolysis occurred when bacteria were incubated in darkness. A later study pursued a similar strategy and employed pDawn in *E. coli* Nissle to drive the expression of α-hemolysin from *Staphylococcus aureus* (Alizadeh et al., 2020). Blue light promoted toxin production and elicited cell lysis on blood agar, whereas in darkness little or no lysis happened. The supernatant of bacterial cultures incubated under blue light triggered apoptosis in colon carcinoma cells, whereas the supernatant of dark-incubated bacteria showed lower propensity for doing so. Although the path towards eventual application will doubtless be long and arduous, therapeutic settings may benefit from the ability to govern cell killing with precise timing, dosing, and spatial control, as enabled by the optogenetic circuits.

Another study used optogenetics to control by light the virulence of *P. aeruginosa via* the YGS24:GacA TCS (Cheng et al., 2021). Blue light promoted the transcription of two endogenous small regulatory RNAs (sRNA) which in turn relieved translational repression of several virulence factors e.g., components of the secretion system. Following ingestion by *C. elegans*, the bacteria resided inside the digestive tract of the animal where they could be activated by blue light. Doing so enhanced the bacterial pathogenicity and caused the killing of the animals. The ability to control pathogenicity of bacteria inside animals paves the way towards kinetic and mechanistic studies of the infection process (Cheng et al., 2021).

Two studies explored the optogenetic manipulation of living materials consisting of *E. coli* bacteria embedded in agarose hydrogels (Sankaran and del Campo, 2019; Sankaran et al., 2019). The pDawn setup activated the expression of a red-fluorescent reporter by blue light, either leading to intracellular protein production or secretion into the extracellular space. In adapted form, this setup also enabled the light-induced production and release of deoxyviolacein (dVio) which exerts antimicrobial and antitumoral activity. To this effect, the pDawn circuit controlled the expression of the four-gene cluster *vioABCE*. Upon blue-light exposure, the living material responded by dVio release; remarkably, the embedded bacteria stayed viable and light-responsive for up to around 40 days. To facilitate light delivery *in situ*, the dVio-secreting composite hydrogels were combined with printed, biodegradable light fibers (Feng et al., 2020). This strategy enabled the optical triggering of the pDawn circuit through several centimeters of animal tissue and may benefit *in vivo* optogenetics in general.

Light-regulated gene expression also served to control bacteria inside the digestive tract of animals. Generally, the pertinent studies leveraged optogenetics to elicit the bacterial production and secretion of substances that bestow benefits on the host. In one example, the CcaRS TCS controlled the expression of the master regulator RcsA in a *E. coli rcsA* knockout strain (Hartsough et al., 2020). Once produced under green light, RcsA activated the expression of the 19-gene *wca* operon for the synthesis and secretion of the exopolysaccharide colanic acid (CA). Notably, CA not only contributes to biofilm formation in *E. coli* and related species, but can also increase the lifespan of *C. elegans* once ingested. In the specific study, *C. elegans* worms were fed bacteria which express RcsA under the CcaRS optogenetic control. Consequently, green light promoted CA production within bacteria residing in the worm intestine and thereby granted the animals longevity. It is worth noting that constitutive CA production, for instance in a bacterial strain lacking the Lon protease, led to similar lifespan increases. Therefore, the primary utility of this captivating study is arguably to be seen in the ability to study the mechanism of the underlying microbe-animal interactions in unprecedented detail. An earlier study (Zhang and Poh, 2018) also targeted CA biosynthesis in *E. coli* by optogenetically controlling the expression of the structural gene *wcaF* within the *wca* operon. To this end, EL222 served as a transcriptional repressor and mediated the transcription in darkness of a gRNA directed against *wcaF*. In combination with dCas9, *wcaF* expression and CA production were repressed in darkness. Blue light suspended gRNA transcription and thus ramped up CA levels, in turn allowing the bacteria to form biofilms. As discussed below, biofilms also underpin the photolithographic production of structured materials.

A slew of studies applied optogenetic expression control in *E. coli* BL21 and the probiotic *L. lactis* and *E. coli* Nissle 1917, with the aim of deploying these bacteria as biotherapeutic agents. Notably, both *L. lactis* and the *E. coli* Nissle strain are suitable for oral administration in microbial therapy. A pioneering report harnessed pDawn to control the expression and subsequent secretion of transforming growth factor β1 (TGF-β1) in *E. coli* BL21 (Yang et al., 2020). To this end, the bacteria were encapsulated and combined with upconverting nanoparticles that emit blue light upon absorption of (several quanta of) NIR light around 980 nm. The UNPs thus enabled activation of the pDawn circuit in bacteria dwelling inside the colon of mice. When this setup was applied in a mouse model of the inflammatory bowel disease ulcerative colitis, the secretion of the cytokine TGF-β1, induced by NIR light, ameliorated the symptoms of the disease. A related study (Cui et al., 2021) also investigated the use of light-responsive bacteria as biotherapeutics for the treatment of ulcerative colitis. To this end, *E. coli Nissle* bacteria expressing the cytokine interleukin 10 (IL-10) from the pDawn plasmid were applied together with UNPs. Inside the mouse intestinal tract, the bacteria were prompted by NIR light to produce and secrete IL-10. In an ulcerative colitis model, this approach reduced the adverse effects of bowel inflammation. By combining the optogenetic therapy with the optical detection of a disease biomarker (i.e., diagnostics), the study advanced a so-called optotheranostic platform. In the future, patients might use this platform to monitor themselves the disease state and progression and to then activate the optogenetic circuit for treatment as required. The earlier study also employed the pDawn circuit in *L. lactis* for the light-induced production of the cytokine interferon γ (IFN-γ) (Yang et al., 2020). Again, the bacteria were encapsulated with UNPs to enable triggering by NIR light. Optogenetically induced IFN-γ secretion in the mouse intestine slowed down progression of a mouse melanoma tumor. More recently, the *L. lactis* strain harboring the pDawn-IFN-γ circuit was used synergistically with photodynamic therapy (PDT) (Wu et al., 2022). To this end, the UNPs were first altered such that they can be excited by irradiation with 808 nm which is less strongly absorbed by tissue than 980-nm light, thus reducing potentially harmful heating. Upon illumination, one sort of UNP reacted by activating a photosensitizer which in turn generated singlet oxygen and other reactive oxygen species for PDT. Another UNP type responded with blue-light emission and thereby elicited IFN-γ secretion by the *Lactococci*. The combination of PDT, optogenetic intervention, and a drug proved most efficient for tumor therapy. The efficiency of the approach was at least partially ascribed to the synergistic activation of the immune system. Along similar lines, NIR light and UNPs activated the expression of the tumor necrosis factor α (TNF-α) from the EL222 circuit in *E. coli* Nissle (Pan et al., 2021). Bacteria harboring this optogenetic circuit were injected into the tail vein of mice together with the UNPs. Subsequent illumination with 980-nm light prompted TNF-α production and greatly decelerated tumor growth in a breast-cancer model.

Light-responsive bacteria were also considered as potential therapeutics for other diseases and disorders. Again with the probiotic *L. lactis* as the chassis, the pDawn circuit was used to control the expression of the hormone glucagon-like peptide 1 (GLP-1) (Zhang et al., 2021, 2022). Upon oral administration, the *Lactococci* stayed viable in the rat intestinal tract for several days. Optogenetic stimulation, either *via* a wearable blue-light-emitting device or *via* UNPs and NIR light, see above, prompted GLP-1 secretion. Notably, GLP-1 enhances the glucose-dependent insulin release by the pancreatic β islet cells, among other effects. In a type-II diabetes rat model, the light-induced GLP-1 production thus lowered the blood glucose levels. Compared to constitutive expression and application of GLP-1, the optogenetically stimulated, intermittent hormone production may reduce the metabolic burden and side effects caused by GLP-1 (Zhang et al., 2021). The application of light-responsive *L. lactis* proved versatile and adaptable to other ends (Pan et al., 2022). By driving the expression of the *gadBC* genes, encoding a glutamate decarboxylase and an antiporter, from the pDawn plasmid, the production of the neurotransmitter γ-aminobutyric acid (GABA) could be activated by UNPs and NIR light. In a mouse model, the light-induced GABA production by orally delivered *L. lactis* reduced anxiety-like symptoms. Moreover, the concentrations of several inflammatory factors in the brain were lowered upon optogenetic stimulation. The underlying strategy also applied to the light-induced production of the granulocyte-colony stimulating factor (GCSF) in *L. lactis* (Pan et al., 2022). In a Parkinson’s disease mouse model, the light-induced GCSF production inside the gut alleviated behavioral symptoms associated with the neurological disorder. Again, the concentrations of inflammatory markers decreased upon the optogenetic intervention.

These equally recent and innovative studies jointly raise the prospect of harnessing light-responsive bacteria as programmable and precisely controllable biotherapeutics. Of key importance, the optogenetic stimulation allows to modulate the response of bacteria inside the digestive tract, bloodstream, or other body compartments of animals. Notably, the relevant use cases to date mostly rely on the blue-light-sensitive pDawn and EL222 systems. Although the limited tissue penetration of blue light was overcome by using UNPs and stimulation with NIR light, this approach incurs potential disadvantages. Specifically, the resultant composite systems are more complex and employ UNPs which as non-biological entities are not genetically encodable and may prove cytotoxic. An alternative route may be the replacement of the blue-light-sensitive optogenetic implements by such that react to light of longer wavelengths, like the CcaRS TCS or the pREDusk/pREDawn circuits.

#### Bacterial photography and structured materials

Applications within this area capitalize on the spatial precision afforded by light-regulated bacterial expression, with some studies exploiting the temporal control in addition (Figure 5C). Early on, researchers realized that optogenetically controlled expression lends itself to the generation of spatial patterns within bacterial communities and lawns. In fact, certain bacteria naturally produce spatially ordered structures when exposed to alternating dark/light cycles e.g., (Kahl et al., 2022). As recently reviewed (Barbier et al., 2022), the patterning of microbial communities gains traction and appears suited for diverse applications in synthetic biology and biotechnology. Although not the only means of organizing bacterial populations in space, see e.g., (Frangipane et al., 2018; Chen and Wegner, 2020; Barbier et al., 2022), light-regulated gene expression appears particularly straight-forward, versatile, and efficient.

Voigt and others pioneered the so-called “bacterial photography” (Levskaya et al., 2005), which has now been demonstrated for many optogenetic circuits e.g., (Levskaya et al., 2005; Ryu et al., 2010, 2014; Tabor et al., 2011; Jayaraman et al., 2016; Romano et al., 2021; Multamäki et al., 2022; Ranzani et al., 2022). Generally, in these applications a bacterial lawn harboring a light-responsive gene expression circuit is exposed to patterned illumination. Owing to differential expression in illuminated and non-illuminated areas, spatial patterns within the bacterial lawn arise and can be visualized *via* suitable reporter genes, mostly fluorescent proteins but also LacZ. The spatial resolution achievable by these photolithographic approaches is principally limited by how well light can be focused and by the size and mobility of individual bacteria within the lawn. To the extent it has been tested, spatial resolution down to micrometer dimensions can be routinely achieved (Ohlendorf et al., 2012; Wang X. et al., 2018; Jin and Riedel-Kruse, 2018; Multamäki et al., 2022). Beyond monochrome systems, the joint use of the Cph8:OmpR and CcaRS TCSs achieved dual-color sensitivity to red/NIR and green/red light, respectively, in *E. coli* cells (Tabor et al., 2011). This strategy was later expanded to RGB sensitivity by adding the YF1:FixJ TCS for sensing blue light (Fernandez-Rodriguez et al., 2017).

In addition to spatially controlling the expression of pigments in microbial communities, patterned structures can also originate from optogenetically modulating bacterial chemotaxis. For instance, both the EL222 (Zhang J. et al., 2020) and the LexRO circuits (Li et al., 2020) served to induce the expression of the bacterial chemotaxis protein CheZ under blue light. Bacteria within illuminated areas hence acquired motility but those in darkness did not. As a corollary, a net movement out of the illuminated areas resulted; put another way, the bacteria underwent negative phototaxis. A conceptually related mechanism for the phototactic movement of *E. coli* was implemented on the basis of the light-driven proton pump proteorhodopsin (Frangipane et al., 2018). Instead of light-dependent gene expression, this fascinating study relied on the light-induced generation of proton motive force across the plasma membrane which powers the bacterial flagellar motor.

Biofilms are widespread among bacteria, and their formation in time and space was repeatedly subjected to optogenetic control, including by light-regulated gene expression. Two studies focused on the so-called antigen 43 (Ag43) (van der Woude and Henderson, 2008), an autotransporter protein exposed on the surface of the outer *E. coli* membrane that mediates intercell contacts and thereby promotes flocculation, aggregation, and biofilm formation (Nakajima et al., 2016a; Jin and Riedel-Kruse, 2018). Using the CcaRS TCS, Ag43 expression in *E. coli* was induced by green light and led to the aggregation and precipitation of the bacteria (Nakajima et al., 2016a). The approach may serve as a means of cell recovery in bioproduction processes. A later study subjected Ag43 expression to blue-light control using the pDawn platform (Jin and Riedel-Kruse, 2018). *E. coli* bacteria responded by biofilm formation in illuminated areas. This photolithographic strategy enabled the printing of biofilms with spatial resolution down to the micrometer range, thus much surpassing most competing methods.

Precisely patterned biofilms not only benefit the study of the underlying biological processes, but also they are attractive for metabolic engineering, diagnostics, and material science. This idea is indeed borne out by a recent study that employed pDawn to photolithographically manufacture biofilms of *Shewanella oneidensis* (Zhao et al., 2022). These facultative anaerobic bacteria are of interest because of their capability of reducing metal ions and forming electrically conductive biofilms. To promote the formation of such films, pDawn drove the expression of different cell-surface proteins engaged in cell-cell interactions. Using the CdrAB proteins from *P. aeruginosa* to this end, the *S. oneidensis* bacteria aggregated under blue light and formed biofilms. Capitalizing on the spatial definition achieved by optogenetics, biofilms of desired extent and specifications could be produced. When inserted between two electrical leads, the biofilm acted as a living electrode material and conducted current. The electrochemical properties of the biofilm could be easily tuned by varying the illumination pattern and duration. An earlier study (Hu et al., 2017) optogenetically drove biofilm formation in *S. oneidensis* as well but used the c-di-GMP-producing BphS to this end. NIR light thus promoted bacterial deposition on an electrode, with scope for potential applications in microbial fuel cells. BphS also enabled the NIR-light-induced formation of *E. coli* biofilms for use as living biocatalysts (Hu et al., 2019). As demonstrated for the conversion of tryptophan into indole within such biofilms, this strategy may apply to bioproduction processes. BphS was further employed for optogenetically modulating biofilms of the opportunistic pathogen *P. aeruginosa* (Huang et al., 2018). To precisely regulate intracellular c-di-GMP levels, BphS was combined with BlrP1 from *K. pneumoniae* which serves as a blue-light-activated EAL phosphodiesterase that degrades this second messenger. Elevated c-di-GMP prompted the expression of several target genes, including *cdrAB*, see above, and thus resulted in biofilm formation. The bimodal regulation by blue and NIR light yielded precisely structured biofilms, well suited to analyzing the transition between planktonic and sessile lifestyles that contributes to the *P. aeruginosa* virulence. In a related approach, the pDawn circuit mediated the expression of a constitutively active EAL enzyme in *P. aeruginosa* (Pu et al., 2018). Within bacteria exposed to blue light, intracellular c-di-GMP was thus depleted and biofilm formation reduced. Finally, EL222 was used to control the *wgaAB* genes in a corresponding *S. meliloti* knockout strain (Pirhanov et al., 2021). Blue light prompted gene expression and thereby activated the biosynthesis of exopolysaccharides which promoted cell aggregation and biofilm formation. *S. meliloti* biofilms of varying extent, thickness, and properties were thus obtained and can be prospectively used for the analysis of plant-rhizobium interactions.

The above use cases compellingly illustrate how light-regulated gene expression can establish spatial patterns in bacterial communities. Beyond their utility in basic research, these approaches garner interest for the production of structured materials, as already hinted at in certain of the above examples (Zhao et al., 2022; Figure 5C). Several studies employed the CsgA protein which upon secretion forms so-called curli fibrils on the bacterial cell surface and mediates biofilm formation (Chen et al., 2014). Importantly, CsgA can accommodate guest peptides and proteins which are thereby displayed on the bacterial outside in high copy number. *Via* the pDawn circuit, the expression of polyhistidine-tagged CsgA was spatially controlled by blue light down to a resolution of around 100 µm (Wang X. et al., 2018). Within the illuminated areas, *E. coli* bacteria thus assembled into biofilms decorated with polyhistidine moieties that can enter metal coordination bonds with certain divalent cations. Using this strategy, diverse nanoobjects linked to nitriloacetic acid tags could be assembled on the biofilm with precise spatial control. Given its modularity, this strategy proved versatile and suitable for different applications. In addition to labeling with fluorescent probes, the light-responsive biofilms could also direct the spatial assembly of gold nanoparticles which upon further derivatization conducted electrical current. Taken together, the light-controlled, programmable assembly of various nanoobjects empowers the hierarchical construction of two- and three-dimensional materials. The principal concept extended to the multimodal optogenetic expression of several CsgA variants bearing different tags (Moser et al., 2019). Based on a previously constructed *E. coli* strain with RGB sensitivity (Fernandez-Rodriguez et al., 2017), the formation and surface properties of biofilms, as well as the protein expression within the constituent bacteria, could be spatially controlled by different light colors. This platform enabled the photolithographic printing of biofilm patterns on several materials, including glass and textiles.

Bacteria that light-dependently express CsgA derivatives were also deployed as living glue systems (An et al., 2020). To this end, the CsgA protein was fused to the Mfp3s peptide which derives from the foot protein of mussels and mediates attachment to solid support. Compared to CsgA alone, expression of the CsgA-Mfp3s protein resulted in stronger adhesion of the bacterial biofilms. The adhesive properties were further increased by including a tyrosinase in the system which catalyzed the oxidation of tyrosine to *ortho*-dihydroxyphenylalanine within the Mfp3s domain. The biofilm could furthermore capture polystyrene microspheres which became entangled within the curli fibrils and formed a strong composite. Light-activated CsgA-Mfp3s expression from the pDawn circuit thus enabled the localized formation of hybrid materials which could serve as glue to repair defects in other materials. This concept was later extended by mineralizing the biofilms with calcium phosphate (Wang et al., 2021). The Mfp3s protein, spread out along the curli fibrils, nucleated the deposition of the salt as hydroxyapatite on the biofilm. By applying different blue-light regimes, the resulting composite material could be adjusted in its spatial extent, thickness, and mechanical properties. Combined with the above microspheres, the living biomaterial formed an even stronger material, designated cement, that again served to repair lesions in materials on demand.

### Perspectives

Following in the footsteps of the first setup for the light-dependent control of bacterial gene expression (Levskaya et al., 2005), numerous optogenetic strategies have been added over the past two decades (see Table 1). As detailed in this article, the presently available repertoire covers the entire near-UV to NIR portion of the electromagnetic spectrum (see Figure 1B). Although mostly based on the regulation of transcription initiation and elongation, several of these systems unlock additional toeholds for the optogenetic expression control, e.g., by acting at the levels of recombination or translation (see Figures 3, 4). After their initial description and proof-of-principle demonstration, at least certain of the available optogenetic tools have stood the test of practice, as they have been adopted for synthetic biology, bioproduction, and theranostics. By capitalizing on the advantages of optogenetics, i.e. genetic encoding, spatiotemporal precision, reversibility, and non-invasiveness, novel applications arose that were previously impossible or even inconceivable (see Figure 5). At the time of writing, the setups based on EL222 (Motta-Mena et al., 2014; Jayaraman et al., 2016), CcaRS (Tabor et al., 2011), and YF1:FixJ (Möglich et al., 2009; Ohlendorf et al., 2012) are the most widely deployed (see Figures 4B,C). In part, this predominance likely owes to legacy, that is, the early implementation of these system. That notwithstanding, numerous examples bridging multiple application areas and bacterial species suggest that these setups offer particularly robust and stringent optogenetic responses in diverse settings.

The flurry of new optogenetic tools released over just the past 2 years e.g., (Li et al., 2020; Sheets et al., 2020; Lalwani et al., 2021a; Dietler et al., 2021; Romano et al., 2021; Multamäki et al., 2022; Ranzani et al., 2022), is testament to the increasing relevance of light-controlled bacterial expression for basic and applied research. With each new setup, key questions beg: Are the already available tools limiting, and if so, how and when? How should and how can they be improved? Which tools are currently lacking for controlling bacterial expression? Although answering these questions universally (and, without bias) is challenging, several trends emerge. First, for at least some of the existing optogenetic implements, there seems to be scope for improvement of, for instance, basal activity, dynamic range, and sensitivity. As admirably demonstrated for the CcaRS TCS (Ong and Tabor, 2018), the unrelenting optimization of the photoreceptor itself and downstream circuitry can greatly boost these parameters. Second, not least given the equally promising and exciting applications of light-regulated bacterial gene expression for theranostics, there appears to be unmet demand for optogenetic circuits that sensitively react to light of long wavelengths, preferably within the so-called NIR transparent window (Weissleder, 2001). Third, notwithstanding the diversity and ingenuity of the arsenal for optogenetic control of bacterial expression, room may exist for additional implements, for instance (d)Cas9 variants that would enable the efficient regulation by light of endogenous genes encoded on the bacterial chromosome. Similarly, other members of the Cas family, such as Cas13 which targets RNA molecules (Abudayyeh et al., 2016), have not been explored for light control so far, but could unlock new directions for the optogenetic control in bacteria and beyond. Future design efforts along all three lines will doubtless benefit from the inherent modularity of many optogenetic circuits. This is equally true for light-responsive TCSs e.g., (Levskaya et al., 2005; Möglich et al., 2009; Ong and Tabor, 2018; Multamäki et al., 2022), and oligomerization-based setups e.g., (Chen et al., 2016; Li et al., 2020; Dietler et al., 2021; Kaberniuk et al., 2021; Romano et al., 2021), either of which can be altered by substituting one photosensor module for another. The latter category stands to benefit from years of basic research and genome mining that provided diverse photoreceptor pairs that associate or dissociate upon light exposure (Strauss et al., 2005; Guntas et al., 2015; Wang et al., 2016; Redchuk et al., 2017; Kuwasaki et al., 2022). Promisingly, recent reports demonstrated the functional expression of plant phytochromes (Raghavan et al., 2020) and cryptochromes (McQuillen et al., 2022) in *E. coli*, thus raising the prospect of bacterial use of these photoreceptors which underpin manifold and highly stringent optogenetic systems in mammalian cells. Furthermore, mechanistic studies provided insights on key residues and structural features which determine the kinetics (Kawano et al., 2013; Pudasaini et al., 2015), direction (Möglich et al., 2009; Ohlendorf et al., 2012; Nakajima et al., 2016b), and dynamic range (Strickland et al., 2010; Gourinchas et al., 2019; Dietler et al., 2021) of photoswitching, thereby facilitating the rational design of photoreceptors and optogenetic circuits with tailored functionality. Libraries of systematically or randomly constructed receptor and circuit variants can be combed by high-throughput screening based on fluorescence signals. Such approaches can complement rational design strategies, especially for complex engineering challenges or when the mechanistic understanding of the target system is limited.

In pursuit of optogenetic control in deep biological tissue, several strategies aim at using red or NIR light to excite photoreceptors. Although residue exchanges around the chromophore could in principle induce a red-shift of absorbance spectra, such efforts had only modest success in flavin-based photoreceptors, arguably owing to the rigid scaffold of the isoalloxazine heterocycle (Goncharov et al., 2021; Röllen et al., 2021). By contrast, the color diversity realized across CBCRs and certain algal phytochromes (Rockwell et al., 2014; Fushimi and Narikawa, 2019) suggests that the chromophore and its absorbance spectrum are more malleable in the phytochrome superfamily. Alternatively, the original chromophore can be substituted for red-shifted versions, provided the bacteria can import or produce the heterologous chromophores (Mathes et al., 2009). However, the incorporation of heterologous chromophores without compromising photochemistry and signal transduction remains a major challenge (Mathes et al., 2009). While point mutations around the chromophore were extensively explored in nature and the lab to create red-shifted channelrhodopsins (Klapoetke et al., 2014), for the optogenetic control of bacterial gene expression the replacement of photosensors for red-light sensitive versions i.e., members of the phytochrome family, turned out to be the more successful strategy (Kaberniuk et al., 2021; Multamäki et al., 2022). Short of photosensor substitution, excitation at double or triple the original wavelength can be achieved by multiple-photon stimulation. Although widely applied in channelrhodopsins (Rickgauer and Tank, 2009), two-photon excitation has to date not seen much use in bacterial optogenetics. Two-photon excitation is principally feasible for at least certain soluble photoreceptor classes (Piatkevich et al., 2017; Homans et al., 2018; Sokolovski et al., 2021), but may suffer from low two-photon absorption cross sections (Kinjo et al., 2019). Nanotechnology offers an alternative approach that generates visible excitation light in deep tissue. For instance, UNPs convert NIR light into shorter wavelengths that in turn activate optogenetic circuits (Hososhima et al., 2015; Chen et al., 2018; Yang et al., 2020). In bigger specimens such as humans or for transcranial stimulation through bone, NIR excitation is still limited to shallow tissue regions. Though not yet applied to bacterial optogenetics, an interesting alternative may be provided by a different type of nanoparticles which can be charged by irradiation with UV light (Wu et al., 2019). Subsequent stimulation of such mechanoluminescent particles with focused ultrasound prompts the emission of light around 470 nm which can be harvested for eliciting optogenetic responses. Employing this sono-optogenetic strategy, the particles may first be charged outside of the animal body or near its surface before circulating in the bloodstream to the desired site. Triggering of the optogenetic circuit is then accomplished *via* application of ultrasound which penetrates biological tissue readily.

On the whole, light-regulated bacterial expression has undergone a rapid transformation over the past 20 years, in terms of both the optogenetic tools and applications at hand. The use cases realized to date incontrovertibly demonstrate the principal feasibility, versatility, and utility of bacterial expression control by optogenetics. Moreover, they are bound to spark additional applications along similar but hopefully also unrelated lines. Researchers pursuing such efforts can choose from and adapt a wide repertoire of options; we hope that the current survey informs this choice.
